# State-dependent promoter switching mediates integrase gene transcription in the integrative and conjugative element ICE*Kp1* of *Klebsiella pneumoniae*

**DOI:** 10.1093/nar/gkag254

**Published:** 2026-03-27

**Authors:** Guitian Liu, Zaizhong Huang, Jiahao Guan, Cui Tai, Lilu Shi, Yanjie Chao, Ruilan Wang, Ke Wang, Hong-Yu Ou

**Affiliations:** State Key Laboratory of Microbial Metabolism, Joint International Laboratory on Metabolic and Developmental Sciences, School of Life Sciences and Biotechnology, Shanghai Jiao Tong University, Shanghai 200240, China; Department of Respiratory and Critical Care Medicine, First Affiliated Hospital of Guangxi Medical University, Nanning 530021, China; State Key Laboratory of Microbial Metabolism, Joint International Laboratory on Metabolic and Developmental Sciences, School of Life Sciences and Biotechnology, Shanghai Jiao Tong University, Shanghai 200240, China; State Key Laboratory of Microbial Metabolism, Joint International Laboratory on Metabolic and Developmental Sciences, School of Life Sciences and Biotechnology, Shanghai Jiao Tong University, Shanghai 200240, China; Department of Respiratory and Critical Care Medicine, First Affiliated Hospital of Guangxi Medical University, Nanning 530021, China; State Key Laboratory of RNA Innovation, Science and Engineering, Vaccine Research Center, Shanghai Institute of Materia Medica, Chinese Academy of Sciences, Shanghai 201203, China; Department of Critical Care Medicine, Shanghai General Hospital, Shanghai Jiao Tong University School of Medicine, Shanghai 200080, China; Department of Respiratory and Critical Care Medicine, First Affiliated Hospital of Guangxi Medical University, Nanning 530021, China; State Key Laboratory of Microbial Metabolism, Joint International Laboratory on Metabolic and Developmental Sciences, School of Life Sciences and Biotechnology, Shanghai Jiao Tong University, Shanghai 200240, China; Shanghai Key Laboratory of Emergency Prevention, Diagnosis and Treatment of Respiratory Infectious Diseases, Shanghai 200025, China

## Abstract

Integrative and conjugative elements (ICEs) are mobile genetic elements that widely disseminate adaptive traits among bacterial populations. Despite their prevalence, the regulatory mechanisms orchestrating ICE integration and excision remain poorly understood. Here, we investigate the transcriptional regulation of the integrase gene (*int*) in ICE*Kp1* of *Klebsiella pneumoniae*. Our results demonstrate that the integrated ICE*Kp1* exploits the adjacent host transfer RNA-*asn* promoter to drive *int* transcription, whereas the excised ICE*Kp1* switches to a 3′-end promoter. Remarkably, the messenger RNA stem-loops within the *int* 5′-UTR are organized as a potential terminator, attenuating the total amount of the full-length *asn*-*int* transcripts during ICE*Kp1*’s integration. Ligation of the 3′ end and 5′ end of ICE*Kp1* maintains the activity of the 3′-end promoter for the *int* transcription in the excised ICE*Kp1*. This promoter switch of the integrase gene promotes a preference for ICE*Kp1* integration over excision. Furthermore, analogous regulatory mechanisms are observed in ICE*clc* and ICE*Ec1*, suggesting evolutionary conservation of this regulatory strategy among diverse ICE families. These findings uncover a state-dependent *int* transcriptional regulatory mechanism that tends ICE toward the integrated state, highlighting the complex interplay between ICEs and their bacterial hosts.

## Graphical Abstract

**Graphical Abstract:**
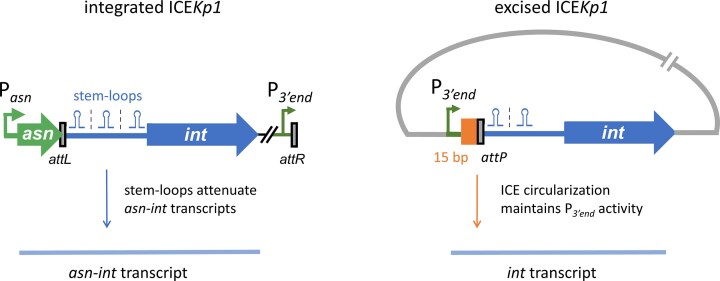

## Introduction

Integrative and conjugative elements (ICEs) are mobile genetic elements crucial for bacterial adaptation and evolution [1]. Over 2000 ICEs have been identified in completely sequenced bacterial chromosomes, with >1000 predicted in human microbiome samples [2]. These self-transferable elements often carry cargo genes conferring antibiotic resistance, virulence traits, and other adaptive functions that enhance host fitness [1, 3]. The interactions between ICEs and host bacteria, particularly regarding transfer regulation, require further investigation [4].

ICEs typically reside in an integrated state within the host chromosome [5, 6]. Upon activation, ICEs can excise, conjugate, and reintegrate [5]. However, excision and conjugation pose risks of ICE loss [5, 7]. Multiple stabilization mechanisms have been identified, including transient replication increasing copy number [8], plasmid-like partitioning systems [9], toxin–antitoxin systems [10], and inhibition of recipient restriction-modification systems [11]. The excised state is rare; for ICE*clc*, only 3%–5% of cells become transfer-competent [12], requiring nutrient induction [7]. Importantly, ICE excision and conjugation imposes cellular burdens; *e.g*., the premature integration of ICE*Bs1* reduces recipient viability [13].

The integration and excision of ICE is mediated by ICE-coding integrase and accessory factors (*e.g*., excisionase). Tight regulation maintains the low-level expression of the integrase gene (*int*) in integrated ICEs, involving ICE-coding specific regulators in ICE*Bs1* [14], ICE*SXT* [15], and Tn*916* [16]. Notably, distinct promoter modalities regulate *int* transcription in the integrated versus excised ICEs. For instance, in *Yersinia pestis* HPI, the *int* transcription start site (TSS) lies upstream of the integration site transfer RNA (tRNA)-*asn* [17], while excised ICE*clc* utilizes a 3′-terminal promoter [18]. Further investigation of these *int* transcriptional mechanisms is warranted.

The clinically significant pathogen *Klebsiella pneumoniae* (including multidrug-resistant and hypervirulent strains) harbors ICE*Kp1*, a 76-kb ICE originally identified in hypervirulent strain NTUH-K2044 [19] (Supplementary Fig. S1G). It encodes important virulence factors, such as yersiniabactin and salmochelin [20]. Its 5′ region shares high similarity with the non-transmissible *Y. pestis* HPI (lacking a type IV secretion system). Given its low-frequency transmission [19], understanding ICE*Kp1*’s integrase gene regulation is particularly important.

In this study, we explored the relationship between the integration tendency of ICE*Kp1* and the transcriptional regulation of the integrase gene. We found that the integrated ICE*Kp1* utilizes the adjacent host tRNA-*asn* promoter (P*_asn_*) for *int* transcription, whereas the excised ICE*Kp1* switches to its’ 3′ terminal promoter (P*_3’end_*). This mechanism reinforces the integration preference of ICE*Kp1*, a conserved feature also observed in ICE*Ec1* and ICE*clc*. These results may advance understanding of ICE regulatory strategies and their implications for bacterial genome plasticity.

## Materials and methods

### Strains, plasmids, and primers

All strains and plasmids used in this study are listed in Supplementary Table S1. All primers used in this study are listed in Supplementary Table S2. All deletion mutants were generated by lambda red recombination using the vector pKOBEG-Apra as previously described [21]. All strains used in this study were cultured at 37°C in lysogeny broth (LB) medium (pH = 7.2–7.4) with appropriate antibiotics.

### Complete genome sequencing and assembly

The genomic DNA of *K. pneumoniae* KpSJTU083 and the four transconjugants that inserted ICE*Kp1* (KpSJTU083C1–KpSJTU083C4) was extracted and sequenced by using the combination of the 2 × 150 bp paired-end Illumina NovaSeq platform and the Oxford Nanopore MinION platform. The resulting filtered reads were then assembled by using Unicycler (v0.5.0) [22] and Flye (v2.9.1-b1781) [23]. Comparative analysis of the genome sequence was performed by using Proksee [24].

### Growth curves and ICE*Kp1* stability determination

Growth curves of NTUH-K2044, NTUH-K2044IT, KpSJTU083, KpSJTU083A, KpSJTU083C1-C4, *Escherichia coli* HB101, *E. coli* HB101::ICE*Kp1, E. coli* C600, and *E. coli* C600::ICE*Kp1* were determined with a Bioscreen C machine. The strains were grown in LB broth at 37°C for 24 h, and the OD_600_ was read automatically every 30 min.

The stability of ICE*Kp1* was determined in NTUH-K2044IT, KpSJTU083C1–C4, *E. coli* HB101::ICE*Kp1*, and *E. coli* C600::ICE*Kp1* as described previously [25], with some modifications. The strains were cultured until the logarithmic growth phase, after which single colonies were selected and subjected to continuous relaxed cultivation in liquid LB medium without antibiotics for 7 days. Subculturing was performed every 24 h. Ten single colonies were selected each time and tested for ICE*Kp1* loss using replica plating on LB agar plates containing 200 µg/ml hygromycin, and on antibiotic-free LB agar plates. For colonies that failed to grow on the hygromycin plates (ICE*Kp1* negative), further confirmation was conducted via polymerase chain reaction (PCR) analysis.

### Quantitative real-time PCR

Total RNA was isolated using the RNeasy Mini Kit (Qiagen). Then, genomic DNA (gDNA) was removed and complementary DNA (cDNA) was produced by *Evo M-MLV* RT Kit with gDNA Clean for qPCR (Accurate Biology, Cat. No.AG11711). Finally, qPCR was performed using the qTOWER^3^G Touch Real-Time PCR System (Analytik Jena), and *gapA* was used as an internal control. The messenger RNA (mRNA) abundance level was calculated by the 2^−ΔΔCT^ method.

### Knockdown the RNase E gene by using the CRISPRi system

Utilizing a CRISPR interference (CRISPRi) system under the control of a *lac* operator [26, 27], we knocked down the expression of the RNase E gene (*rne*) in KpSJTU083C1-C4 (Fig. 2E–G and Supplementary Fig. S5). One millimolar isopropyl-3-D-thiogalactopyranoside (IPTG) induces system expression to block target gene transcription via protein–RNA–DNA complex formation, achieving gene knockdown; target genes are transcribed normally without IPTG. Strains used included *E. coli* WM3064 [DAP auxotrophic, harboring RP4 (tra)], WM3064 derivatives with pJMP2844 (single guide RNA (sgRNA) cloning, a gift from Jason Peters; Addgene plasmid #160675; http://n2t.net/addgene:160675;  RRID: Addgene_160 675) [27] or pJMP1039 (helper plasmid, a gift from Carol Gross & Jason Peters & Oren Rosenberg; Addgene plasmid #119239; http://n2t.net/addgene:119239;  RRID: Addgene_119239) [26], and KpSJTU083C1-C4.

Target-specific spacers were generated by annealing (37°C for 30 min, 95°C for 3 min, slow cooling) with T4 PNK, T4 PNK buffer, Spacer-F/R, and nuclease-free water. pJMP2844 was digested with BsaI (37°C for 90 min) and ligated with diluted spacers (room temperature, 90–120 min). Ligation products were transformed into WM3064 competent cells (ice incubation, 42°C heat shock, DAP-supplemented LB recovery), followed by plating, colony sequencing, and verification.

Conjugation was performed by activating donor (pJMP2844-spacer WM3064), helper (pJMP1039 WM3064), and recipient strains, culturing to OD_600 _= 0.5, mixing equally, centrifuging, resuspending, and spotting on DAP-supplemented LB plates (30°C, overnight). Bacterial lawns were resuspended, diluted, and plated on chloramphenicol-resistant plates (no DAP). Positive transconjugants were confirmed by PCR for sgRNA insertion and preserved.

We designed specific primers for the junction sequence of *asn*-*int* (Fig. 2F) as well as *argX-hisR*, a previously documented co-transcript [28] used as a control (Supplementary Fig. S5A). The cleavage of RNase E was quantified by measuring the mRNA abundance of the junction sequence. After IPTG induction, the mRNA level of the *rne*-targeting dCas9 was increased (Fig. 2G). In contrast, the mRNA abundance of the *rne* was decreased. The mRNA abundance of the *asn*-*int* and *argX*-*hisR* co-transcript increased (Fig. 2G and Supplementary Fig. S5B). This result confirmed the functionality of the *rne*-knockdown CRISPRi system in KpSJTU083C1-C4.

### β-galactosidase assay

The β-galactosidase activity was quantified based on the hydrolysis of O-nitrobenzene-β-D-galactopyranoside (ONPG). Bacteria were subcultured in LB broth to logarithmic phase, then cells were pelleted and resuspended in an equal amount of Z buffer (0.06 M Na_2_HPO_4_, 0.04 M NaH_2_PO_4_, 0.01 M KCl, 0.001 M MgSO_4_, 0.05 M β-mercaptoethanol). OD_600_ was measured blank against the Z buffer. One milliliter cell was permeabilized by adding 100 μl chloroform and 50 μl 0.1% sodium dodecyl sulfate and equilibrated for 5 min in a 28°C water bath. The reaction was started by adding 0.2 ml ONPG (4 mg/ml) at 28°C and was stopped after a sufficient yellow color had developed by adding 0.5 ml 1 M Na_2_CO_3_. The reaction time (*T*) was recorded precisely with a timer. Then the mixture was spined 5 min at maximum to remove debris and chloroform. OD_420_ and OD_550_ of the supernatant were measured. Units of activity were calculated with the formula as follows:


\begin{eqnarray*}
\mathrm{Miller}\ \mathrm{Units} = 1000\ \times {\mathrm{\ }}\frac{{(\mathrm{ O}{{\mathrm{ D}}_{420}} - \mathrm{ O}{{\mathrm{ D}}_{\mathrm{ 550}}}){\mathrm{\ }} \times {\mathrm{\ }}1.75}}{{T\ \times \ \mathrm{ O}{{\mathrm{ D}}_{600}}}}.
\end{eqnarray*}

### Luciferase assay

The luciferase assay was employed for promoter activity determination. Prepare a 6× luciferase solution with a concentration of 2.76 mg/ml as the substrate for the luciferase assay. Inoculate bacteria into a medium composed of 30 μl of substrate mixed with 150 μl of LB and culture until the logarithmic growth phase. Measure the bacteria’s OD_600_ value and luciferase activity using the Varioskan LUX plate reader (Thermo Scientific), and calculate the relative light units (RLU) with the following formula:


\begin{eqnarray*}
\mathrm{ RLU} = \frac{{{\mathrm{luciferase\ activity}}}}{{\mathrm{ OD}_{ 600}}}.
\end{eqnarray*}

### 5′ rapid amplification of cDNA end assay

The total RNA was prepared with the RNAprotect Bacterial Reagent and RNeasy Mini Kit (Qiagen, Germany, Cat. No. 74104) following the manufacturer’s instructions. The 5′ rapid amplification of cDNA end (5′-RACE) assay was performed using SMARTer® RACE 5′/3′ Kit (Takara, Clontech) with some modifications. In the rapid amplification of cDNA ends, we substitute the SeqAmp DNA Polymerase with *AdeptTect* Flash HS PCR Master Mix (dye plus) (Accurate Biology, Cat. No. AG12301). The PCR program followed the manufacturer’s instructions.

### Conjugation assay

The conjugation assay was performed as described previously [29], with minor modifications. *Klebsiella pneumoniae* NTUH-K2044IT (resistant to hygromycin) was employed as a donor strain. KpSJTU083A (KpSJTU083-pACYC184-apr, resistant to apramycin) was employed as a recipient. The donor and the recipient were cultured in LB media at 220 rpm and 37°C for 12 h. Then, the donor cells and the recipient cells were recovered by centrifugation (9000 rpm, 10 min). After that, resuspend the pellet in 1 ml phosphate buffered saline twice, then resuspend the donor cells in 10 mM MgSO_4_ (20 μl). Mix the donor cells and the recipient cells sufficiently and transfer 20 μl of the mixture on LB agar. Incubate at 37°C for 24 h. After the conjugation, LB agar with 200 µg/ml hygromycin and 50 µg/ml streptomycin or 50 µg/ml apramycin was used for the selection of transconjugants. The transconjugants were further validated by PCR. The conjugation frequency was calculated as the ratio of transconjugants to donors. All conjugation experiments were conducted in three biological replicates with three parallel tests.

### Detection of the excision of ICE*Kp1* by droplet digital PCR

The primer pairs were designed to target the *attL, attR, attB*, and *attP* sites, with Taqman probes located at the 3′ end of the forward primers (Supplementary Table S2). The probes were labeled with a FAM fluorescent reporter group at the 5′ end and a BHQ1 quencher group at the 3′ end. KpSJTU083C1–C4 was cultured to the logarithmic growth phase, after which genomic DNA was extracted and quantified to a concentration of 16 ng/μl using NanoDrop. The DNA sample was then diluted 10 000-fold to a concentration of 1.6 × 10^−3^ ng/μl for the detection of the *attL* and *attR* sequence copy numbers by droplet digital PCR (ddPCR), while undiluted DNA samples (16 ng/μl) were used for the detection of the *attB* and *attP* sequence copy numbers. The ddPCR was performed using the Naica Crystal Digital PCR system with Sapphire chips, following the manufacturer’s instructions for the PCR program. The excision frequency of ICE*Kp1* was calculated with the formula as follows:


\begin{eqnarray*}
\textit{ICEKp}1\ \mathrm{excision}\ \mathrm{frequency} = {\mathrm{\ }}\frac{{2 \times \textit{attB}}}{{\textit{attL} + \textit{attR} + \left( {2 \times \textit{attB}} \right)}} \times {{10}^{ - 4}}.
\end{eqnarray*}

### Analysis of the transcription start site and the promoter of the excised *int*-ICE*Kp1*

To identify the TSS of the excised *int*-ICE*Kp1*, we firstly cloned the integrase gene, ICE*Kp1* 5′ (161 bp) and 3′ ends (604 bp) into the high-copy number plasmid, pBluescript SK(+). Then, a 5′-RACE assay was performed to map the TSS of the excised *int*-ICE*Kp1* (5′/3′-RACE Kit, Accurate Biology, Cat. No.AG11618). Next, potential promoters of the *int*-ICE*Kp1*, based on the TSS of the excised *int*-ICE*Kp1*, were predicted by using BPROM [30] and SAPPHIRE [31]. Finally, the potential promoters were verified by point mutations and the β-galactosidase assay.

### Statistical analysis

Student’s *t*-test (unpaired, equal variance, two-sided) and ordinary one-way analysis of variance were performed using the R package (https://www.r-project.org/). Statistical significance was considered when *P* ≤ .05. ns indicates not significant.

## Results

### Excision and conjugation events of ICE*Kp1* are infrequent in its lifecycle

**Figure 1.**
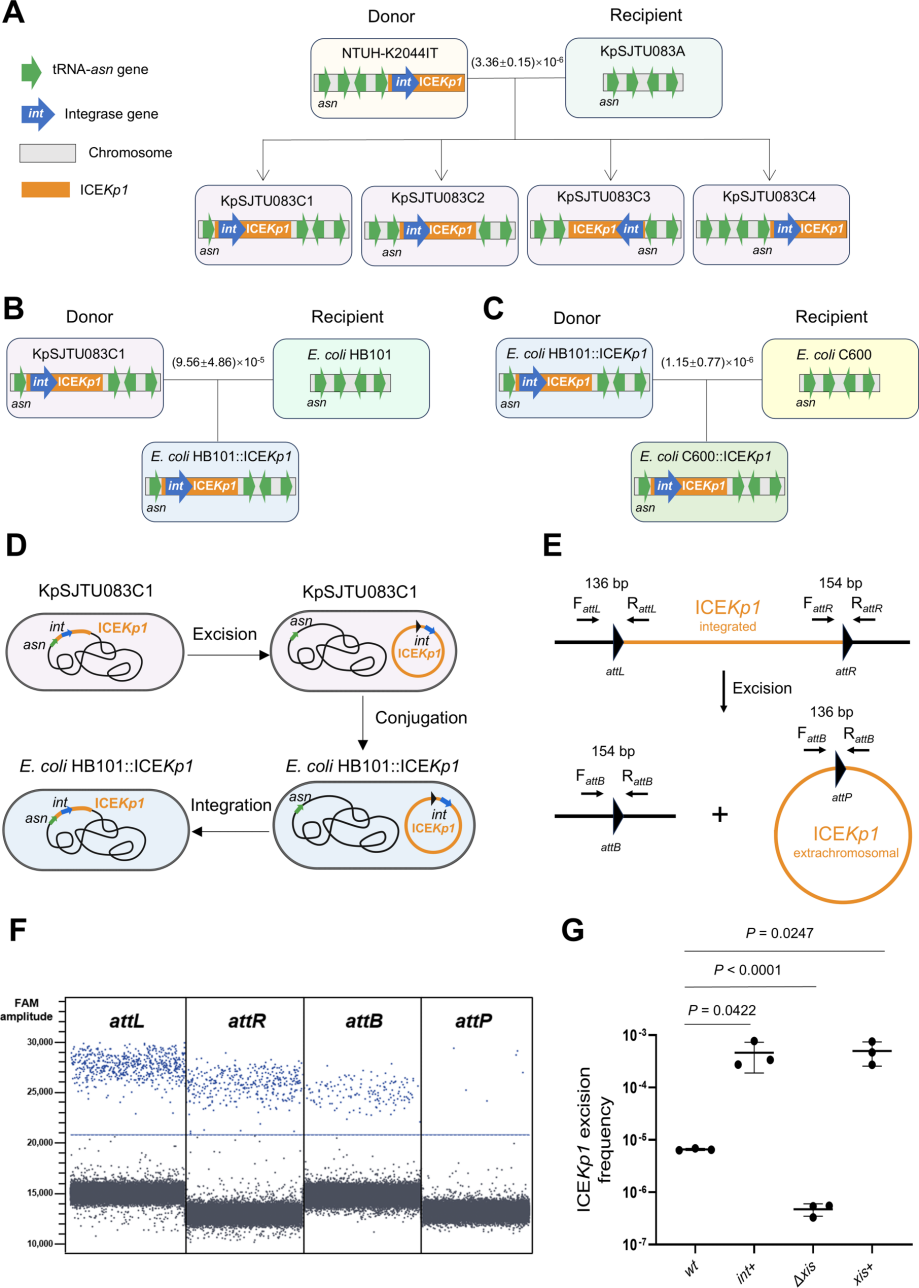
ICE*Kp1* excision level correlated with integrase gene expression in *K. pneumoniae*. Conjugation of ICE*Kp1* from *K. pneumoniae* NTUH-K2044IT to *K. pneumoniae* KpSJTU083A (**A**), *K. pneumoniae* KpSJTU083C1 to *E. coli* HB101 (**B**), and *E. coli* HB101::ICE*Kp1* to *E. coli* C600 (**C**). (**D**) Schematic map of ICE*Kp1* conjugation process from *K. pneumoniae* KpSJTU083C1 to *E. coli* HB101. (**E**) Schematic map of the excision of ICE*Kp1* and the primer design strategy for testing ICE*Kp1* excision by ddPCR. (**F**) The 1D droplet spots of FAM fluorescence amplitude of *attL, attR, attB*, and *attP* sites detection in KpSJTU083C1. Each point denotes a droplet, and those above the blue threshold line are classified as positive (blue points) (data of KpSJTU083C2–C4 available in Supplementary Fig. S3). (**G**) Correlation between ICE*Kp1* excision frequency and expression levels of integrase gene (*int*) and excisionase gene (*xis*).

To assess the dissemination potential of ICE*Kp1*, we performed conjugation transfer assays within *K. pneumoniae* (intraspecies) and between *K. pneumoniae* and *E. coli* (interspecies). In the first round of the conjugation assay, we observed the transfer of ICE*Kp1* from the donor strain *K. pneumoniae* NTUH-K2044IT (derived from NTUH-K2044, knocked in the hygromycin resistance *hph* in ICE*Kp1*) to the recipient strain *K. pneumoniae* KpSJTU083A (derived from KpSJTU083, introduced a plasmid pACYC184-apr carrying the apramycin resistance gene), occurring at a low frequency of (3.36 ± 0.15) × 10^−6^ (Fig. 1A). To detect the integration of ICE*Kp1* in KpSJTU083A, we performed tRIP-PCR [32] on the four tRNA-*asn* genetic loci (named *asn1* to *asn4*) on the chromosome of KpSJTU083A (Supplementary Fig. S1A). All four tRNA-*asn* sites were found to be the integration sites for ICE*Kp1* (Fig. 1A and Supplementary Fig. S1B). Four types of transconjugants were obtained (KpSJTU083C1, KpSJTU083C2, KpSJTU083C3, and KpSJTU083C4). This was further verified by the whole-genome sequencing of the four transconjugants, which supported the integration of ICE*Kp1* into the given tRNA-*asn* site (Supplementary Fig. S1C and D). The analysis of growth curves and the ICE*Kp1* maintenance test of KpSJTU083C1-C4 (Supplementary Fig. S1E and F) indicated that the integration of ICE*Kp1* did not impose a heavy metabolic burden on the host strain and could stably reside in the host chromosome.

To investigate the transfer of ICE*Kp1* in *E. coli*, a second round of the conjugation assay was conducted, utilizing KpSJTU083C1–C4 as donors and *E. coli* HB101 as recipient (Fig. 1B and Supplementary Fig. S2A). This demonstrated that ICE*Kp1* transferred from *K. pneumoniae* to *E. coli* at a low frequency approaching 10^−5^ (Fig. 1B and Supplementary Fig. S2A). In the third round of the conjugation assay, ICE*Kp1* subsequently transferred from *E. coli* HB101 to *E. coli* C600 at a frequency of ~10^−6^ (Fig. 1C). The assessment of growth curves and ICE*Kp1* maintenance tests for *E. coli* HB101::ICE*Kp1* and *E. coli* C600::ICE*Kp1* (Supplementary Fig. S2B and C) indicated that ICE*Kp1* was reliably inherited in non-native hosts without imposing a significant metabolic burden.

The above results demonstrated that ICE*Kp1* is capable of conjugative transfer both intraspecifically and interspecifically, at a low frequency. Since ICEs need to excise from the chromosome prior to conjugative transfer (Fig. 1D), we hypothesize that the low-frequency conjugative transfer of ICE*Kp1* may be attributed to its low excision frequency. Therefore, we detected the excision frequency of ICE*Kp1* in KpSJTU083C1–C4. The excision of ICE*Kp1* was detected by using ddPCR (Fig. 1E and F; Supplementary Fig. S3A–C), and the excision frequency was calculated based on the copy number of *attB* (see the “Materials and methods” section for details). The results showed that the excision frequency of ICE*Kp1* in KpSJTU083C1–C4 was low as (3.72 ± 0.57) × 10^−5^, (1.18 ± 0.07) × 10^−4^, (8.43 ± 1.18) × 10^−5^, and (4.16 ± 1.24) × 10^−5^, respectively, which was comparable to its conjugative transfer frequency (Fig. 1B and Supplementary Fig. S2A). ICEs typically undergo site-specific recombination under the catalysis of their encoded integrases (*int*) and accessory factors (*e.g*., excisionases, *xis*), thereby excising from the chromosome. Therefore, *int* and *xis* expression levels correlate strongly with ICE excision frequency. Previous studies showed that the knockout of the integrase gene of ICE*Kp1* abolished its excision and conjugation [19]. To further investigate the effects of *int* and *xis* on the excision frequency of ICE*Kp1*, we separately performed *int* overexpression, *xis* knockout, and *xis* overexpression, followed by the detection of ICE*Kp1* excision frequency. The results showed that the *int* overexpression by itself could significantly enhance the excision frequency of ICE*Kp1* (Fig. 1G). In addition, overexpression of *xis* on its own also enhanced ICE*Kp1* excision frequency. Since *int* and *xis* of ICE*Kp1* are located on separate transcripts (Supplementary Fig. S1G), this indicates that their expression may be subject to complex regulation. Collectively, these findings suggest that the transcriptional regulation of the *int* restricts the excision of ICE*Kp1* and further impairs its conjugative transfer capability. On this basis, we subsequently investigated the transcriptional regulatory mechanism of the ICE*Kp1* integrase gene.

### Integrated ICE*Kp1* displaces the terminator of the adjacent tRNA-*asn* gene and exploits its promoter for the integrase gene transcription

**Figure 2.**
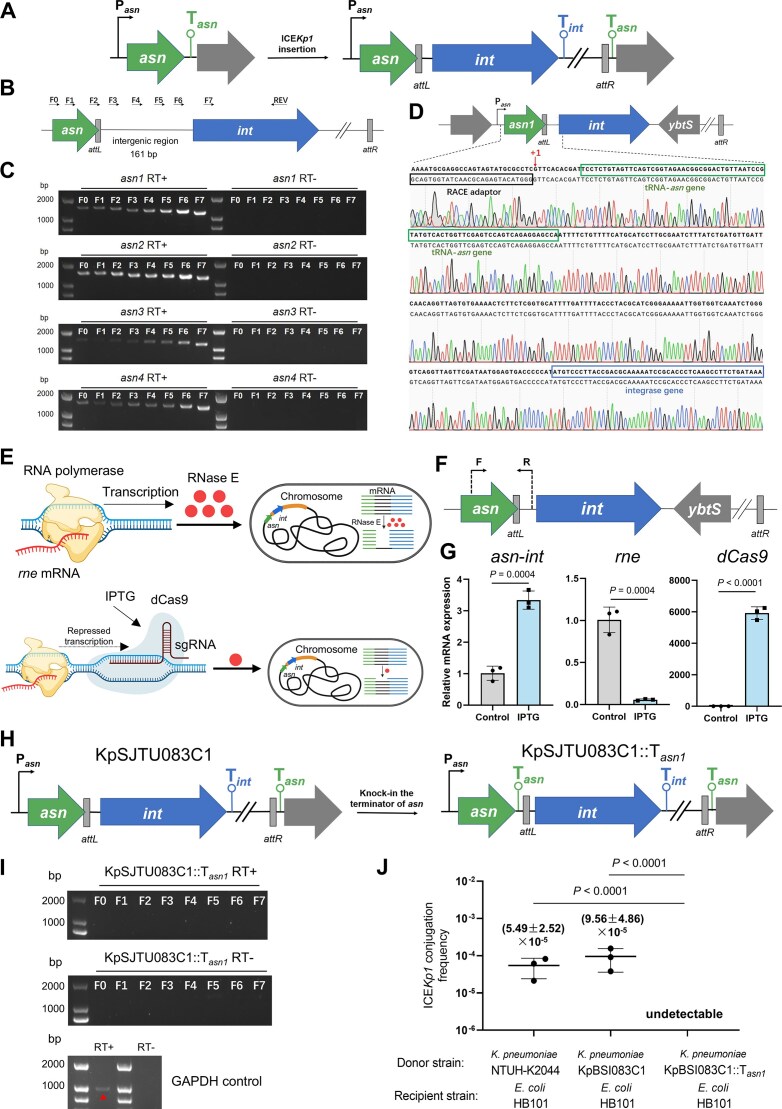
ICE*Kp1* displaces the tRNA-*asn* terminator and exploits the tRNA-*asn* promoter to initiate *int*-ICE*Kp1* transcription in the integrated state. (**A**) Schematic map of the location change event of the tRNA-*asn* terminator by ICE*Kp1* insertion. (**B**) Schematic map of the *asn*-*int* polycistronic operon. F0–F7 are forward primers (from the TSS to *int*) used in the reverse-transcription PCR (RT-PCR). REV is the reward primer used in the RT-PCR. (**C**) Co-transcription of the *asn*-*int* operon in KpSJTU083C1–KpSJTU083C4 verified by RT-PCR. (**D**) TSS of *int* was identified in KpSJTU083C1 by 5′-RACE experiment. Sequence alignment was performed between the tRNA-*asn1* gene (*asn1*) promoter (P*_asn_*) to *int* (top) and the sequence identified by the 5′-RACE experiment (bottom). “+1” refers to the TSS of *int* (data of NTUH-K2044 and KpSJTU083C2–C4 available in Supplementary Fig. S4). (**E**) Schematic map of the cleavage of the *asn*-*int* co-transcript detected by CRISPRi. Intact lines (green part was the tRNA-*asn* region, black part was the intergenic region, and the blue part was the *int* region) refer to the intact *asn*-*int* transcripts. Broken lines refer to the *asn*-*int* transcript cleaved by RNase E. (**F**) Schematic map of the primer design for identification of the *asn*-*int* co-transcript cleavage. (**G**) mRNA abundance of the *asn*-*int* transcript junction sequence, *rne* and *dCas9*. The mRNA quantity was normalized against the housekeeping gene *gapA*. (**H**) Schematic map of the tRNA-*asn* terminator inserted in KpSJTU083C1. (**I**) The co-transcription of *asn*-*int* operon in KpSJTU083C1::T*_asn1_* verified by RT-PCR. Transcript of *gapA* was used as the control. (**J**) ICE*Kp1* transfer frequency determined between NTUH-K2044, KpSJTU083C1, and KpSJTU083C1::Tasn1 (donor strains) with *E. coli* HB101 (recipient strain).

To investigate the limited transferability of ICE*Kp1*, we focused on the transcription of its integrase gene (*int-*ICE*Kp1*) located at the 5′ end of ICE*Kp1*. This gene is positioned 161 bp downstream of ICE*Kp1*’s insertion site, the tRNA*-asn* gene, and is transcribed in the same direction. In the ICE*Kp1*-free strain KpSJTU083, a Rho-independent transcription terminator (T*_asn_*) is located at the 3′ end of the empty tRNA-*asn* gene. However, upon ICE*Kp1* integration at its 3′ end (KpSJTU083C1-C4), T*_asn_* was displaced to the downstream of the 3′ end of ICE*Kp1*, resulting in the loss of its function to terminate transcription of tRNA-*asn* (Fig. 2A). Therefore, we hypothesize that in the integrated state of ICE*Kp1, int-*ICE*Kp1* is co-transcribed with the upstream tRNA-*asn* gene in the host chromosome, resulting in the formation of a polycistronic transcript designated as *asn-int*.

First, we examined the TSS of the *int-*ICE*Kp1* for the integrated ICE*Kp1* using the reverse transcriptase (RT)-PCR and the assay of 5′-RACE assay. The RT-PCR results (Fig. 2B and C) showed that, in KpSJTU083C1–C4, *int-*ICE*Kp1* and the adjacent tRNA-*asn* were co-transcribed. Results of 5′-RACE further pinpointed the TSS of the *int-*ICE*Kp1* to be located 11 bp upstream of the first base pair of the tRNA-*asn* gene (*asn1, asn2*, or *asn3*) in KpSJTU083C1–C3, and 3 bp upstream of the first base pair of *asn4* in KpSJTU083C4 and NTUH-K2044 (Fig. 2D and Supplementary Fig. S4A–D).

Next, to further verify the co-transcription of *asn*-*int*, we examined the cleavage of the *asn*-*int* co-transcript by RNase E (Fig. 2E and F). After IPTG-induced activation of the CRISPRi system targeting *rne* in KpSJTU083C1–C4, the significant increase in the mRNA abundance of the *asn*-*int* junction sequence revealed that the *asn*-*int* co-transcript was susceptible to cleavage by RNase E in KpSJTU083C1-C4 (Fig. 2G and Supplementary Fig. S5).

Finally, we conducted a genetic knock-in of T*_asn_* at the 3′ end of the tRNA-*asn* gene in KpBSI083C1 (KpBSI083C1::T*_asn1_*) to disrupt the formation of *asn*-*int* co-transcription (Fig. 2H). The RT-PCR results demonstrated that the tRNA-*asn* gene and the *int-*ICE*Kp1* could not form a co-transcript in KpBSI083C1::T*_asn1_* (Fig. 2I). In addition, a conjugation assay was performed to assess the potential transfer of ICE*Kp1* from KpBSI083C1::T*_asn1_* to *E. coli* HB101. As anticipated, no transconjugants were obtained in this assay (Fig. 2J).

Together, these results demonstrated that the integration of ICE*Kp1* induced the displacement of T*_asn_*, thereby exploiting the tRNA-*asn* promoter to initiate the transcription of *int-*ICE*Kp1* during the integrated state of ICE*Kp1*. It supports the hypothesis that the promoter of the tRNA-*asn* (P*_asn_*) on the host chromosome initiates the transcription of the *int-*ICE*Kp1*, resulting in the production of *asn*-*int*.

### Excised ICE*Kp1* exploits the 3′ end promoter for the integrase gene transcription

**Figure 3.**
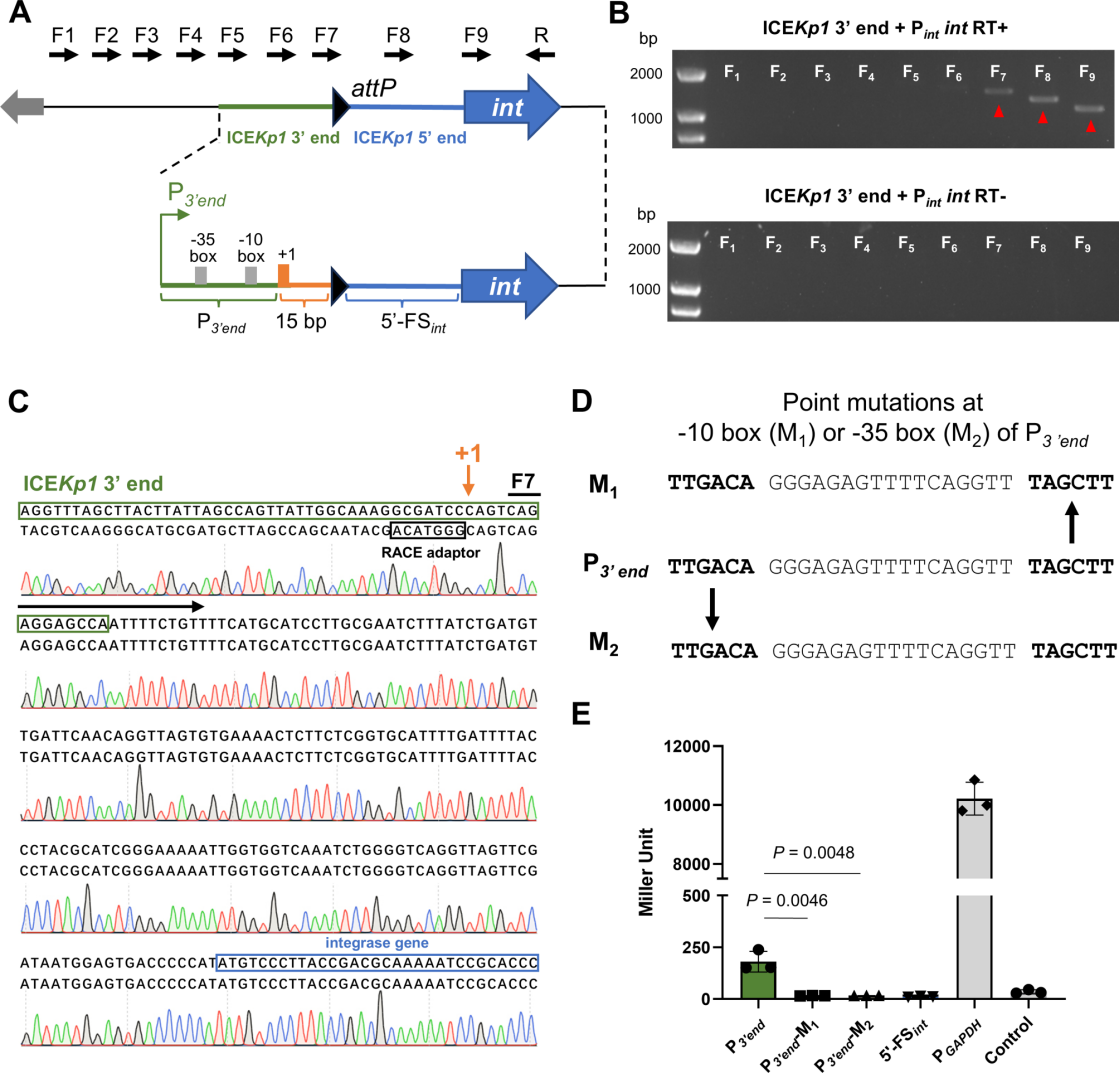
Excised ICE*Kp1* exploits the 3′ end promoter for the integrase gene transcription. (**A**) Schematic map of the primer design strategy for detection of *int* transcript by RT-PCR and the *int* promoter at the excised state, “+1” represents the TSS of the *int*. (**B**) Transcript of *int* was detected by RT-PCR at the ICE*Kp1* excised state. (**C**) TSS of *int* was identified by 5′-RACE experiment in the excised state. Sequence alignment was performed between the ICE*Kp1* 3′ end to *int* (top) and the sequence identified by the 5′-RACE experiment (bottom). “+1” refers to the TSS of *int*. (**D**) Schematic map of the P*_3’end_* point mutations at the −10 box (M_1_) or −35 box (M_2_). (**E**) The promoter activities of the P*_3’end_* and its point mutants were determined by β-galactosidase assay.

In its excised state, ICE*Kp1* exists as an extrachromosomal entity, with the joining of its 3′ and 5′ end occurring at the *attP* site (Fig. 3A). Sequence alignment analyses indicate that the 3′ and 5′ ends are highly conserved among ICE*Kp1*-like elements (Supplementary Figs S6 and S7). A putative σ^70^ promoter (P*_3’end_*) was identified in proximity to the 3′ end, and we hypothesize that this promoter may initiate the transcription of *int*-ICE*Kp1* in the 5′ end of the excised ICE*Kp1*.

First, the TSS of *int*-ICE*Kp1* within the excised ICE*Kp1* was examined through RT-PCR and 5′-RACE assay. Nine forward primers were designed to bind to various locations around the 3′ and 5′ ends, along with a reverse primer specific to the *int-*ICE*Kp1*, facilitating the amplification of cDNA (Fig. 3A). Analysis through RT-PCR (Fig. 3B) and 5′-RACE (Fig. 3C) demonstrated that transcription of *int*-ICE*Kp1* initiated from the 3′ end of the excised ICE*Kp1*. The TSS of the *int-*ICE*Kp1* was located 15 bp upstream of the first nucleotide of the 5′ end. Then, a β-galactosidase assay was conducted to evaluate the promoter activity of the P*_3’end_* and the 5′-FS*_int_* (5′-flanking sequence of the integrase gene). The assay results (Fig. 3E) indicated that the 5′-FS*_int_* region exhibited no promoter activity. In contrast, P*_3’end_* and FL_excised_ (the full-length fragment covering tRNA-*asn* promoter, tRNA-*asn*, and 5′-FS*_int_*) showed detectable promoter activity, suggesting the capacity of P*_3’end_* to drive the transcription of the integrase gene. Finally, specific mutations were introduced into the P*_3’end_* region to further investigate its promoter activity (Fig. 3D and E). Results from β-galactosidase assays analyzing mutations within the −35 and −10 boxes corroborated the hypothesis that the P*_3’end_* is crucial for *int* transcription (Fig. 3E).

Collectively, these findings suggest that a promoter located at the ICE*Kp1* 3′ end, and ICE*Kp1* exploits this promoter to drive the transcription of the integrase gene when in the excised state.

### The stem-loops within the *int* 5′-UTR attenuate the total amount of the full-length *asn-int* transcripts in the integrated ICE*Kp1*

Within the integrated state of ICE*Kp1*, the transcription levels of the tRNA-*asn* gene may lead to a corresponding increase in the transcription of *int*-ICE*Kp1*, potentially resulting in the frequent excision of ICE*Kp1*. This conflicts with the previously observed low excision and transfer frequencies of ICE*Kp1*, as well as the documented minimal expression of the HPI-carrying integrase gene [33]. Therefore, we propose that ICE*Kp1* may down-regulate the activity of the tRNA-*asn* promoter, thereby modulating the transcription of *int*-ICE*Kp1* and facilitating its sustained integration within the host genome. To investigate this hypothesis, we conducted a β-galactosidase assay to evaluate the promoter activity in the upstream region of *int*-ICE*Kp1*.

**Figure 4.**
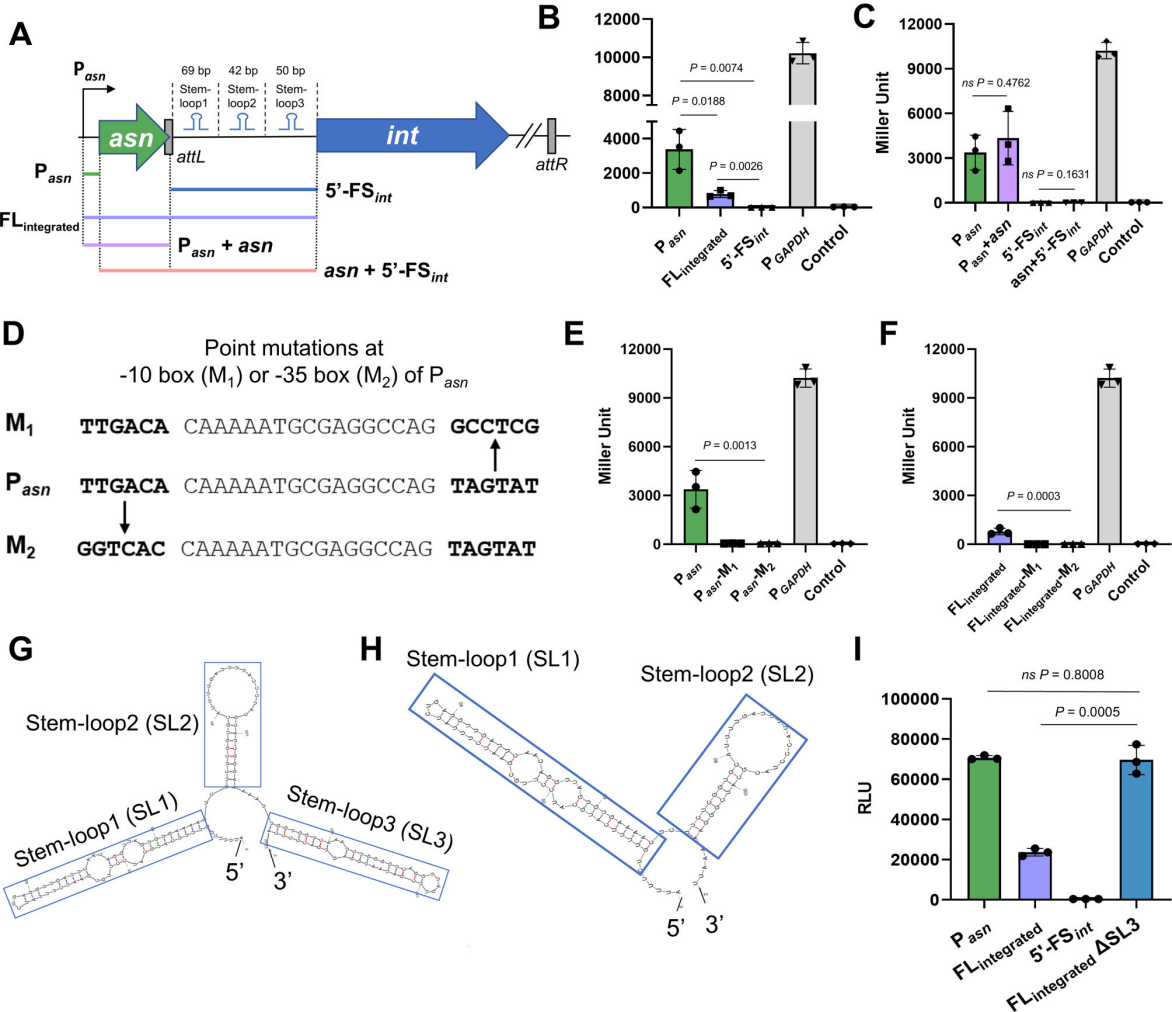
Stem-loops within the 5′ UTR of *int* attenuates the promoter activity of the full-length fragment from P*_asn_* to *int* 5′-UTR in the integrated ICE*Kp1*. (**A**) Schematic map of the upstream fragment of *int* in the ICE*Kp1* integrated state for promoter activity analysis. From the promoter of the tRNA-*asn* gene to the 5′ end of *int*, five groups were set: P*_asn_* (tRNA-*asn* promoter), 5′-FS*_int_* (the 5′ flanking sequence of *int*), FL_integrated_ (the full-length fragment covering tRNA-*asn* promoter, tRNA-*asn* and 5′-FS*_int_*), P*_asn _*+ *asn* (tRNA-*asn* promoter and the tRNA-*asn* gene), and *asn *+ 5′-FS*_int_* (the tRNA-*asn* gene and the 5′-FS*_int_*). (**B, C**) Promoter activities of P*_asn_*, FL_integrated_, 5′-FS*_int_*, P*_asn _*+ *asn*, and *asn *+ 5′-FS*_int_* determined by β-galactosidase assay (data about P*_asn2_*, P*_asn3_*, and P*_asn4_* available in Supplementary Fig. S8). (**D**) Schematic map of the tRNA-*asn1* promoter point mutations at the −10 box (M_1_) or −35 box (M_2_). The point-mutated promoter activity of the P*_asn_* (**E**) and FL_integrated_ (**F**) determined by β-galactosidase assay. “−M_1_” and “−M_2_” refer to “point mutation at −10 box” and “point mutation at −35 box,” respectively. (data about *asn2, asn3*, and *asn4* available in Supplementary Fig. S8). (**G**) The predicted stem-loops in the 5′ flanking region of ICE*Kp1*. (**H**) The predicted mRNA secondary structure after knocking out the stem-loop3 in the 5′ flanking region of ICE*Kp1*. (**I**) Under the integrated state of ICE*Kp1*, the promoter activity of P*_asn_*, FL_integrated_, 5′-FS*_int_*, and FL_integrated_ ΔSL3.

Upon the ICE*Kp1* integrated state, we segmented the upstream region of the *int-*ICE*Kp1* into five distinct fragments (Fig. 4A): P*_asn_* (promoter of the tRNA-*asn* gene), 5′-FS*_int_* (5′ flanking sequence of *int-*ICE*Kp1*), FL_integrated_ (full-length fragment; spanning from P*_asn_* to 5′-FS*_int_* in the integrated state of ICE*Kp1*), P*_asn _*+ *asn* (the tRNA-*asn* gene sequence and its promoter), and *asn *+ 5′-FS*_int_* (the tRNA-*asn* gene sequence and the 5′ flanking sequence of *int-*ICE*Kp1*). The results from the β-galactosidase assay (Fig. 4B) showed that 5′-FS*_int_* exhibited negligible promoter activity, while P*_asn_* demonstrated high activity levels, indicating that the integrase gene exploits P*_asn_* for transcription during the integrated state, whereas the 5′-FS*_int_* region is incapable of driving integrase gene transcription. Moreover, we performed point mutations on the −35 and −10 boxes of P*_asn_* and assessed the activity of P*_asn_* and FL_integrated_ after the mutations (Fig. 4D). The results of the β-galactosidase assay (Fig. 4E and F) showed a decrease in promoter activity for the mutation of FL_integrated_, which correlated with the decline observed in the mutation of P*_asn_*. Similar findings were noted for KpSJTU083C2–C4 (Supplementary Fig. S8).

In comparison to P*_asn_*, FL_integrated_ showed comparatively reduced promoter activity, suggesting that ICE*Kp1* may exert a down-regulatory effect on P*_asn_*. In addition, the promoter activities of P*_asn _*+ *asn* and *asn *+ 5′-FS*_int_* were similar to those of P*_asn_* and 5′-FS*_int_*, respectively (Fig. 4C), suggesting the diminished promoter activity of P*_asn_* was not attributable to the tRNA-*asn* sequence. Thus, the reduced promoter activity of P*_asn_* is likely caused by the 5′-FS*_int_* region in the *int* 5′-UTR. This region harbored three stem-loop (SL) structures as predicted by Mfold [34] (Fig. 4G). We hypothesized that these SL structures might exert a suppressive effect on the promoter activity of P*_asn_*. After confirming that the knockout of individual SL structures did not induce alterations in other secondary structures within the 5′-FS*_int_* region (Fig. 4H and Supplementary Fig. S9A and B), to explore the regulatory role of the SL structures, we generated their knockouts sequentially and evaluated the consequent changes in FL_integrated_ promoter activity by using a luciferase assay (Fig. 4I and Supplementary Fig. S9C–F). The results showed that the deletion of SL3 led to a 194.2% enhancement in promoter activity compared with FL_integrated_, indicating that it exerted a significant inhibitory effect on the promoter activity of P*_asn_* (Fig. 4I). SL1 and SL2 also exert an inhibitory effect on P*_asn_*, albeit to a relatively weaker extent (Supplementary Fig. S9C–F). Together, these findings indicated that the step-loops (SL1–SL3) within the 5′-FS*_int_* region of the *int* 5′-UTR are organized as a potential terminator, which attenuates the total amount of the full-length *asn-int* mRNA transcripts during the integrated state of ICE*Kp1*, thereby maintaining ICE*Kp1* in an integrated state.

### Ligation of the 3′ end and 5′ end of ICE*Kp1* maintains the activity of the 3′ end promoter in the excised ICE*Kp1*

**Figure 5.**
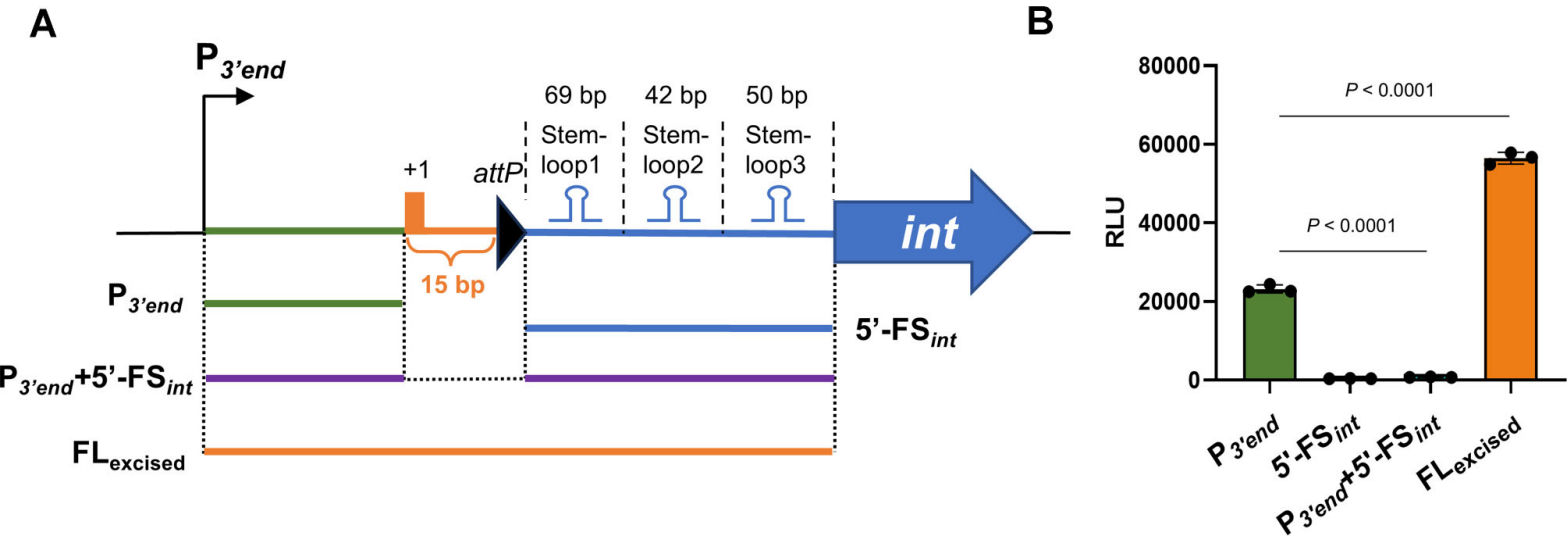
Ligation of the 3′ end and 5′ end of ICE*Kp1* maintains the activity of the 3′ end promoter in the excised ICE*Kp1*. (**A**) Schematic map of the upstream fragment of *int* in the ICE*Kp1* excised state for promoter activity analysis. From the P*_3’end_* to the 5′ end of *int*, four groups were set: P*_3’end_* (the promoter located at the 3′ end of ICE*Kp1*), 5′-FS*_int_* (the 5′ flanking sequence of *int*), P*_3’end _*+ 5′-FS*_int_* (a fusion of the P*_3’end_* sequence and the 5′ flanking sequence of *int-*ICE*Kp1* by deleting 15-bp sequence at the 3′ end of ICE*Kp1*), FL_excised_ (the full-length fragment covering P*_3’end_*, ICE*Kp1* 3′ end from the TSS of *int* to *attP* and 5′-FS*_int_*). (**B**) Under the excised state of ICE*Kp1*, the promoter activity of P*_3’end_*, 5′-FS*_int_*, P*_3’end _*+ 5′-FS*_int_*, and FL_excised_.

Upon the ICE*Kp1* excised state, we analyzed the upstream region of the *int-*ICE*Kp1* by dividing it into four distinct fragments (Fig. 5A): P*_3’end_* (the promoter located at the 3′ end of ICE*Kp1*), 5′-FS*_int_* (the 5′ flanking sequence of *int-*ICE*Kp1*), P*_3’end _*+ 5′-FS*_int_* (a fusion of the P*_3’end_* sequence and the 5′ flanking sequence of *int-*ICE*Kp1* by deleting 15-bp sequence at the 3′ end of ICE*Kp1*), and FL_excised_ (the full-length fragment). The luciferase assay results (Fig. 5B) showed that P*_3’end_* exhibited strong activity, whereas 5′-FS*_int_* displayed weak promoter activity, indicating that ICE*Kp1* exploits P*_3’end_* to drive the transcription of the integrase gene during the excised state. Notably, ligating P*_3’end_* (in the 3′ end of ICE*Kp1*) to 5′-FS*_int_* (in the 5′ end of ICE*Kp1*) to form the full-length fragment (FL_excised_) kept robust promoter activity (Fig. 5B). However, deletion of the 15-bp sequence at the 3′ end of ICE*Kp1* from FL_excised_ abolished promoter activity in the resulting fragment (P*_3’end _*+ 5′-FS*_int_*) (Fig. 5B). This suggests that the 15-bp sequence might counteract the inhibitory effect of 5′-FS*_int_*, which contains stem-loop structures (SL1–SL3) that likely function as a transcriptional terminator (Fig. 5B).

To further investigate whether the 15-bp sequence at the 3′ end of ICE*Kp1* can relieve terminator repression in 5′-FS*_int_*, we conducted the luciferase assay using a heterologous promoter (P*_gapA_*). As shown in Supplementary Fig. S12, 5′-FS*_int_* suppressed P*_gapA_* activity, whereas introducing the 15-bp sequence upstream of 5′-FS*_int_* restored P*_gapA_* activity, suggesting that this 15-bp sequence alleviates terminator repression.

Together, these results indicate that specific regulatory elements within the *int* 5′-UTR sustain P*_3’end_* activity, which may promote the reintegration of excised ICE*Kp1* into the host chromosome.

### A similar state-dependent promoter switching mechanism for the integrase gene transcription is observed in other ICEs

We conducted further investigations to determine whether the *int* promoter activity alteration, observed in the integrated or excised ICE*Kp1*, occurs in other ICEs that possess integrase genes located at their ends, adjacent to host tRNA genes.

**Figure 6.**
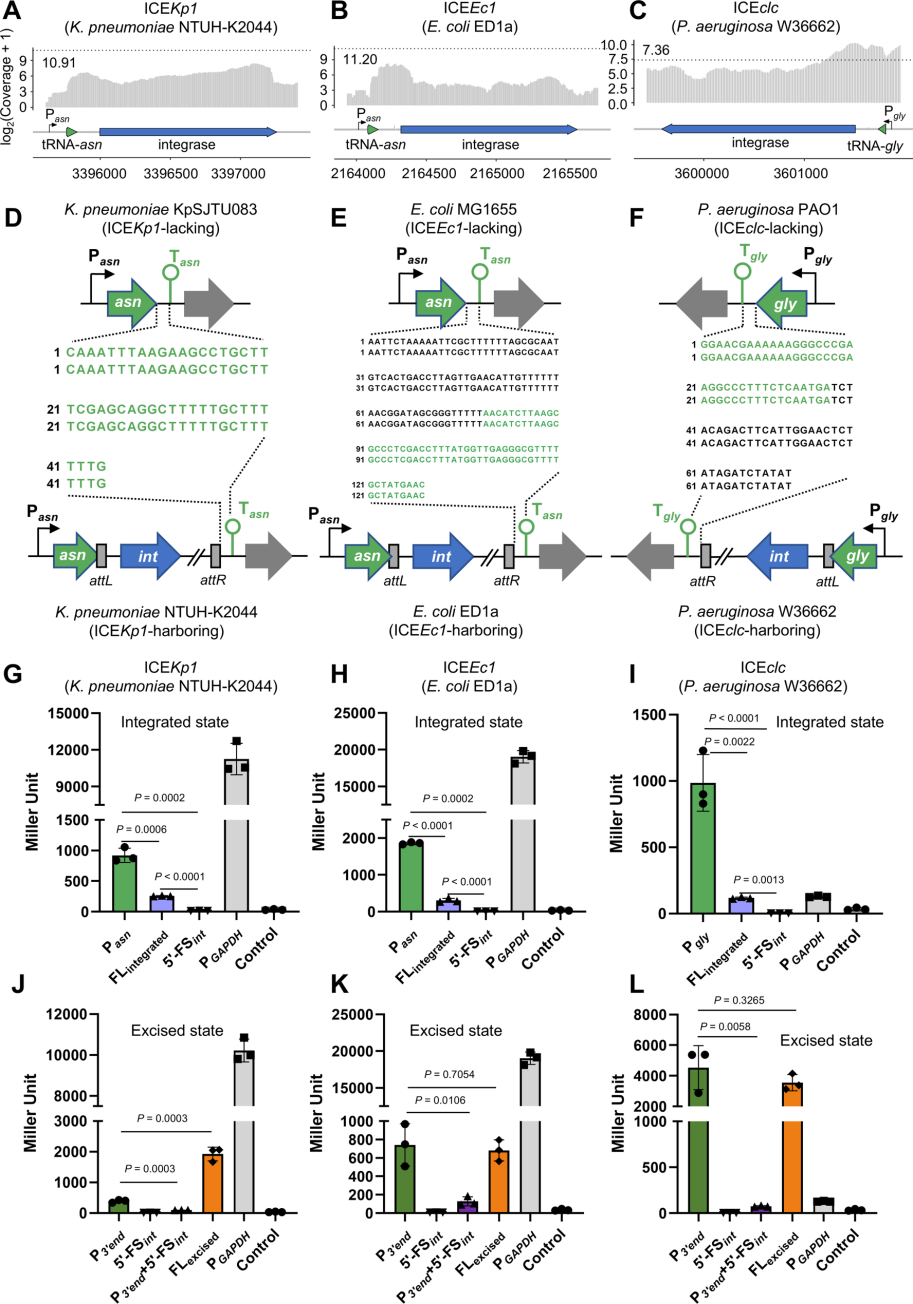
State-dependent promoter switching mediates *int* transcription in other ICE families. (**A**–**C**) “*tRNA*-*int*” co-transcription analysis through the RNA-seq data. The *Y*-axis represents the read coverage of the tRNA gene and *int* in the RNA-seq data. The *X*-axis represents the gene locus of the tRNA gene and *int*. The RNA-seq data were taken from NCBI SRA with the accession numbers: SRX10030082 for *K. pneumoniae* NTUH-K2044, SRX5332356 for *E. coli* ED1a, and SRX12812169 for *Pseudomonas putida* W36662. (**D**–**F**) Relocation of the tRNA gene terminator after ICE insertion. Alignment of the sequence from the 3′ end of the tRNA gene to the terminator without ICE insertion (top) against the sequence of the ICE 3′ end after ICE insertion (bottom). The sequence in green denotes that of the tRNA gene terminator. (**G**–**I**) Promoter activities of the P*_tRNA_*, FL_integrated_, and 5′-FS*_int_* determined by β-galactosidase assay in the ICE integrated state. (**J**–**L**) Promoter activities of the P*_3’end_*, 5′-FS*_int_*, P*_3’end _*+ 5′-FS*_int_*, and FL_excised_ determined by β-galactosidase assay in the ICE excised state.

First, we conducted an *in-silico* analysis examining the co-transcription of the ICE-carrying integrase gene (*int*) and the neighboring tRNA gene in the integration of ICE. From a total of 851 ICEs compiled in the ICEberg database [2], we selected 23 ICEs, each of which integrates at the 3′ end of the tRNA gene and possesses the *int* gene adjacent to the tRNA gene in the same transcriptional strand (see Supplementary Table S3). We subsequently retrieved five ICEs with RNA-seq data available in the NCBI SRA database, including the ICE*Kp1* of *K. pneumoniae* NTUH-K2044, ICE*Ec1* of *E. coli* ED1a, ICE*clc* of *Pseudomonas aeruginosa* W36662, ICE*Ec1* of *E. coli* MS14387, and ICE*Ri1* (ICE*Rin*ATCC49129) of *Ralstonia insidiosa* ATCC 49129 (Supplementary Table S3). Mapping the short reads to the genomic context of the tRNA gene and its adjacent integrase gene (Fig. 6A–C and Supplementary Fig. S10) indicated that these ICEs can leverage the promoters of the tRNA-*asn* genes to initiate transcription of the integrase genes upon the ICE integration into the host chromosome. Additionally, we analyzed the tRNA gene terminator adjacent to the *int* gene within these five ICE-harboring bacterial hosts (Supplementary Table S4). In contrast to ICE-containing hosts, we identified a terminator present at the 3′ end of the tRNA gene in hosts lacking ICEs (Fig. 6D–F and Supplementary Fig. S11). Upon integration of the ICE at the tRNA gene site, the terminator was displaced to the 3′ end of the ICE (Fig. 6D–F and Supplementary Fig. S11).

Next, we performed a β-galactosidase assay to investigate the promoter activity within the upstream region of the *int* of the integrated ICE, with the examples of ICE*Ec1* of *E. coli* ED1a and ICE*clc* of *P. aeruginosa W36662*. The DNA fragments corresponding to the tRNA gene promoter (P*_tRNA_*), the flanking sequence of the integrase gene (5′-FS*_int_*) and the full-length (FL_integrated_) region of ICE*Ec1* and ICE*clc* were synthesized for analysis. The β-galactosidase assay performed on these various fragments of ICE*Ec1*, ICE*clc* and ICE*Kp1* in *K. pneumoniae* NTUH-K2044 (served as a control) revealed that P*_tRNA_* exhibited strong activity while 5′-FS*_int_* demonstrated weak activity. The FL_integrated_ fragment exhibited medium activity in comparison (Fig. 6G–I). The 5′-FS*_int_* in the *int* 5′-UTRs of ICE*Ec1* and ICE*clc* are composed of multiple stem-loop structures, which are similar to those of ICE*Kp1*, as identified by mRNA secondary structure analysis (Supplementary Fig. S13A and C). These findings suggest that the integration of ICE*Ec1* and ICE*clc* induces transcription termination downstream of the stem-loop structures, resulting in the reduction of full-length mRNA transcripts that contain both the tRNA gene and *int*.

Finally, we conducted a β-galactosidase assay to assess the transcriptional activity of the promoter within the upstream region of the *int* of the excised ICE*Ec1* and ICE*clc*. The DNA fragments corresponding to the P*_3’end_* (promoter located at the 3′ end of the ICE), 5′-FS*_int_*, P*_3’end _*+ 5′-FS*_int_*, and FL_excised_ (full-length fragment) of ICE*Ec1* and ICE*clc* were also synthesized for further examination. The results of the β-galactosidase assay indicated that P*_3’end_* and FL_excised_ exhibited strong activity, whereas 5′-FS*_int_* demonstrated weak promoter activity (Fig. 6J–L).

Collectively, the data suggest that ICE*clc* and ICE*Ec1* can switch the *int* promoter upon their integration or excision, and this modulation could facilitate their integration into bacterial host genomes.

## Discussion

The ICE-encoded integrase leads to the integration and excision of ICEs via site-specific recombination [17, 35–38]. However, the factors contributing to the limited transfer frequency of ICEs, particularly in relation to integrase gene expression, remain ambiguous. In this study, we proposed a promoter switching regulation mechanism of the integrase gene transcription that occurs upon the integrated or excised state of ICEs (Graphical abstract). Our investigation concentrates on the ICE*Kp1* family, widespread within the hypervirulent and classical *K. pneumoniae* strains. Notably, ICE*Kp1* demonstrated the capability for self-transfer between *K. pneumoniae* and *E. coli*, occurring at a low frequency (Fig. 1). For other ICE*Kp* families in *K. pneumoniae*, their 3′ and 5′ end sequences are highly conserved relative to ICE*Kp1* (Supplementary Figs S6 and S7), which suggests that this regulatory pattern for the integrase gene is widely prevalent across the ICE*Kp* families. Given that most ICEs lack a replicon and cannot sustain stable existence outside the chromosome, enhanced expression of *int* during the excised state is advantageous for facilitating the re-integration of ICE into the donor chromosome.

The promoter for the integrase gene of non-transmissible HPI of *Y. pestis* was found to be located near the host tRNA-*asn* [17]. In this study, we also found that the integrated ICE*Kp1* exploits the adjacent tRNA-*asn* promoter (P*_asn_*) for the *int* gene (Fig. 2). We further observed that the stem-loops located at the *int* 5-UTR might be organized as a potential terminator, diminishing the total amount of the *asn*-*int* mRNA transcripts (Fig. 4), and ensuring that the transcription of *int* remains at a low level, thereby maintaining the stability of ICE*Kp1* within the host chromosome. The molecular mechanism requires further investigation. Similar insulating gene expression is also observed in Tn*916* [39], indicating that this mechanism may be present in multiple ICE families.

Following excision from the host chromosome, the left end (3′ end) and right end (5′ end) of ICE may recombine at the *attP* site, converting the ICE into a circular form. Within the ICE*clc* family, the *int* gene is located at the 5′ end, adjacent to the ICE*clc* integration site, the tRNA-*gly* gene. During the excised state of ICE*clc*, the promoter P*_cir_*, located at the 3′ end, joins the 5′ end to initiate the *int* transcription [18]. Similarly, we identified a promoter (P*_3’end_*) for the integrase gene at the 3′ end of the excised ICE*Kp1*, located upstream of the *attP* site (Fig. 3). Further investigation observed that the 3′ end of the excised ICE*Kp1* ligated to its 5′ end, generating a full-length fragment (FL_excised_) with robust promoter activity (Fig. 5). The regulatory role of the *int* 5′-UTR appears intricate and remains to be explored in future studies. For example, the *in silico* analysis of the *int* 5′-UTR secondary structure indicated that the 15-bp sequence at the 3′ end of ICE*Kp1* might be able to base-pair with the stem-loop SL3 in 5′-FS*_int_*, potentially destabilizing SL3 and relieving its inhibitory effect on the P*_3’end_* (Supplementary Fig. S13). Future studies should experimentally validate this proposed RNA–RNA interaction between the 15-bp sequence and SL3 to confirm its regulatory role.

tRNA genes are highly conserved, with their 3′ ends frequently serving as integration sites for various ICEs, such as tRNA-*asn* for the ICE*Kp* family of *K. pneumoniae*, tRNA-*gly* for the ICE*clc* family of *P. aeruginosa*, and tRNA-*asn* for the ICE*Ec*1 family of *E. coli*. Following integration into the host chromosome, the integrase gene, positioned at the 5′ end of the ICE, is located ~200 bp away from the integrated tRNA gene (Supplementary Table S3). Through comparative analyses of the tRNA gene terminators, the transcriptomic data of the ICE host strain, and the promoter activities of ICE*cl*c and ICE*Ec1* (Fig. 6), we observed a similar modulation in promoter activity that influences the *int* transcription upon the integration or excision of ICE*Kp1*. This observed phenomenon may extend to additional ICE families, for example, ICE*Ri1* (ICE*Rin*ATCC49129) of *R. insidiosa* (Supplementary Fig. S10).

The transcriptional regulation of the ICE integrase gene involves a complex network, likely mediated by multiple ICE-encoded regulators [14–16, 40]. Differential expression of these regulators between donor and recipient strains may lead to variations in integrase transcription, requiring further study. Additionally, ICE excision frequency is influenced not only by integrase regulation but also by environmental factors such as UV light and stress conditions [41, 42]. Since *K. pneumoniae* (the host of ICE*Kp1*) is an opportunistic pathogen, assessing ICE*Kp1* excision and transfer in *in vivo* animal models would better reflect its real-world transmission dynamics, warranting further investigation.

In summary, we proposed that the state-dependent switching of the integrase gene promoter, promoting integration over excision of ICE*Kp1*. This regulatory mechanism depends on ICE*Kp1’*s state (Graphical abstract): (i) Integrated state: ICE*Kp1* displaces the terminator of the adjacent tRNA-*as*n gene and hijacks its promoter (P*_asn_*) to drive *int* transcription. The mRNA stem-loop structures in the *int* 5′-UTR reduces the full-length *asn*-*int* transcripts. (ii) Excised state: ICE*Kp1* utilizes its 3′ end promoter (P*_3’end_*) for *int* transcription. The *int* 5′-UTR extends into a 15-bp region at the ICE’s 3′ end, which possesses complex mechanisms to keep P*_3’end_* activity. We propose that other ICEs carrying the integrase genes adjacent to host tRNA genes (*e.g*., ICE*clc* and ICE*Ec1*) may employ analogous strategies. Given that the integrase gene is pivotal role in the ICE lifecycle, these findings could advance understanding of how ICEs balance stable maintenance and dissemination in bacterial hosts under stress.

## Supplementary Material

gkag254_Supplemental_File

## Data Availability

The genome sequences of *K. pneumoniae* KpSJTU083 and the transconjugants KpSJTU083C1–KpSJTU083C4 were deposited in the NCBI GenBank repository, with the accession number CP180482, CP180483, CP180377, CP180314, and CP180315.
