# Supplementary material for: State-dependent promoter switching mediates integrase gene transcription in the integrative and conjugative element ICEKp1 of Klebsiella pneumoniae
Source: Nucleic Acids Res. 2026 Mar 27;54(6):gkag254. doi: 10.1093/nar/gkag254 (PMC13023037; doi:10.1093/nar/gkag254)
Supplement: gkag254_Supplemental_File [file gkag254_supplemental_file.pdf]

Supplementary Data

Figure S1. Detection of ICE*Kp1* integration and stability in KpSJTU083C1–C4.

Figure S2. Transfer of ICE*Kp1* across different species.

Figure S3. The 1D droplet spots of FAM fluorescence amplitude of *attL*, *attR*, *attB* and *attP* sites detected in KpSJTU083C2, KpSJTU083C3 and KpSJTU083C4.

Figure S4. Transcription start site of the integrase gene was identified in NTUH-K2044, KpSJTU083C2, KpSJTU083C3 and KpSJTU083C4 by 5'-RACE experiment.

Figure S5. mRNA abundance of the junction sequence in the polycistronic transcripts KpSJTU083C1-KpSJTU083C4.

Figure S6. Sequence alignment and conservation analysis of the *attL* site, 5'-FS_{int} and integrase sequences of ICE*Kp* families (ICE*Kp1*-ICE*Kp14*).

Figure S7. Sequence alignment and conservation analysis of the 3' end of ICE*Kp* families (ICE*Kp1*-ICE*Kp14*).

Figure S8. The promoter activity of the different upstream fragments of the integrase gene determined in the *asn2*, *asn3* and *asn4* groups by β-galactosidase assay.

Figure S9. Detection of the effect of 5'-FS_{int} stem-loop structures on promoter activity.

Figure S10. Analysis of the "*tRNA*-*int*" co-transcription in ICE*Ec1* and ICE*Ri1* by using RNA-seq data.

Figure S11. Location change event of the tRNA gene terminator after inserting different ICE families.

Figure S12. Effects of the FS_{int} and the 15 bp sequence of the ICE*Kp1* 3' end on the activity of the heterologous promoter P_{gapA}.

Figure S13. The predicted mRNA secondary structure of the 5'-FS_{int} and 5' UTR of the *int* in excised ICE*Kp1*, ICE*Ec1* and ICE*clc.*

Table S1. Strains and plasmids used in this study

Table S2. Primers used in this study

Table S3. 23 ICEs which the integrase gene was located at the 5' end of the ICE and no other genes located between the tRNA gene and the integrase gene

Table S4. *In silico* analysis of the ICE-inserted tRNA gene terminator in ICE-harboring and ICE-lacking strains

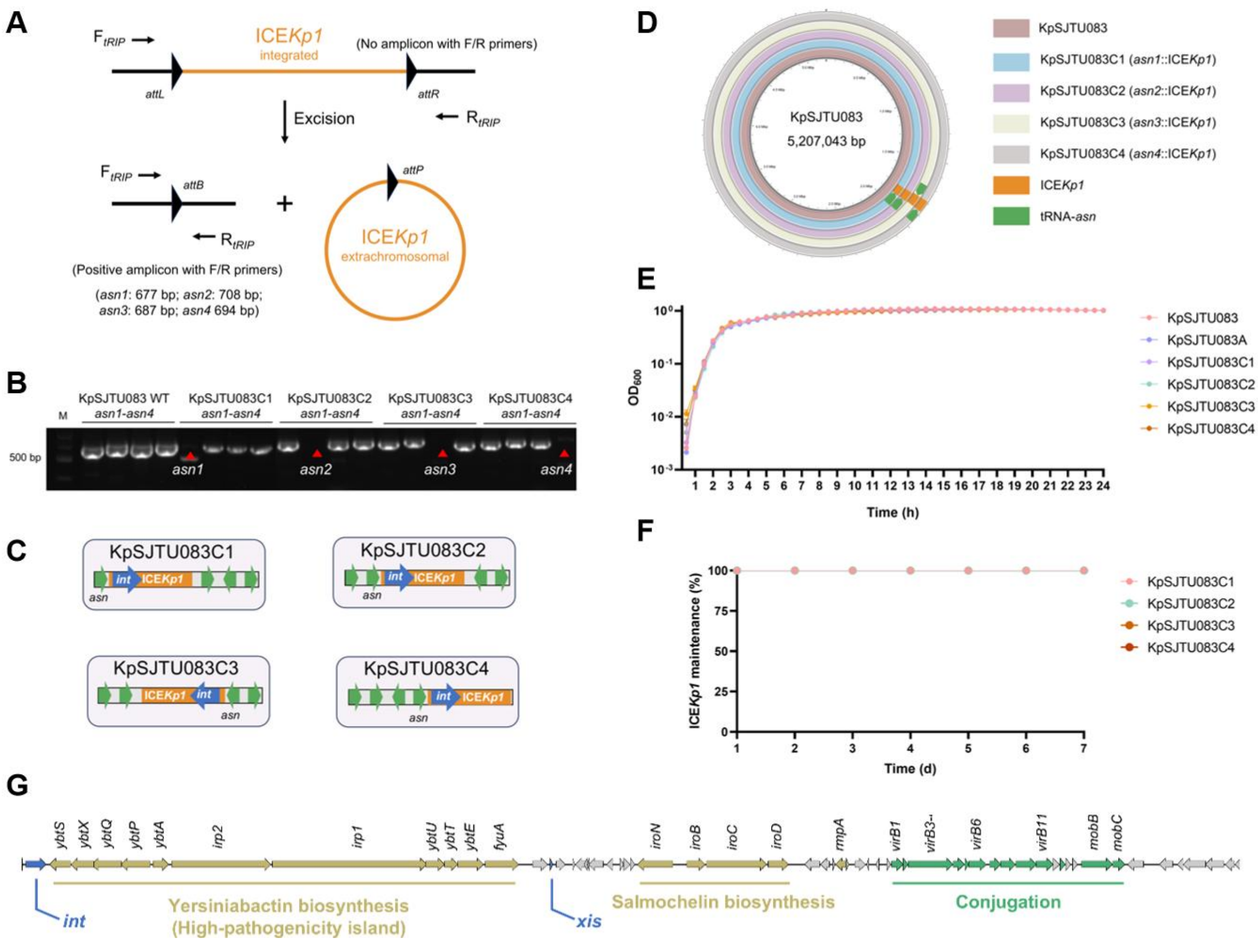

Figure S1. Detection of ICE*Kp1* integration and stability in *K. pneumoniae* KpSJTU083C1–C4. **A.** Schematic map of tRIP-PCR. **B.** Examination of four tRNA-*asn* sites in the wild type KpSJTU083 and four transconjugants KpSJTU083C1-C4 by tRIP-PCR. **C.** Schematic map of the ICE*Kp1* insertion sites in the transconjugants KpSJTU083C1-KpSJTU083C4. **D.** Genome sequence alignments of the individual transconjugants (KpSJTU083C1, KpSJTU083C2, KpSJTU083C3, and KpSJTU083C4) against the recipient strain (KpSJTU083). Circular genome diagram. Orange, ICE*Kp1*. Green, tRNA-*asn* gene. **E.** Growth curves of KpSJTU083, KpSJTU083A and their respective ICE*Kp1* conjugants. **F.** Analysis of the stability of ICE*Kp1* within transconjugants KpSJTU083C1 to KpSJTU083C4. **G.** Genetic map of ICE*Kp1* in *K. pneumoniae* NTUH-K2044. The integrase gene (*int*) and the excisionase gene (*xis*) are marked in blue, the virulence factor genes (*ybt*, *iro* and *rmpA*) are marked in yellow, and the conjugation module (*virB and mob*) are marked in green.

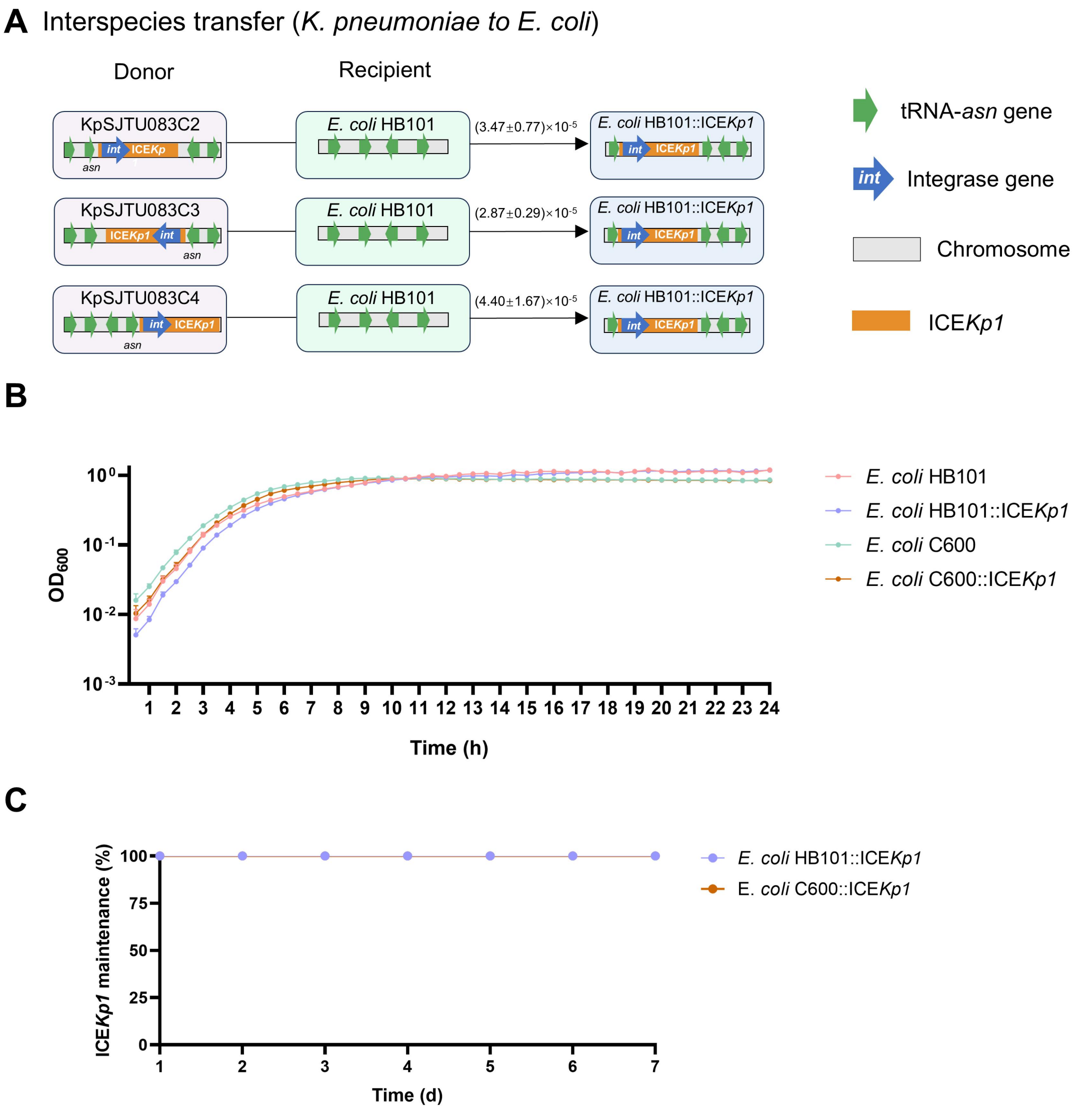

Figure S2. Transfer of ICE*Kp1* across different species. **A.** Interspecies transfer of ICE*Kp1* (*K. pneumoniae* to *E. coli*). Conjugation experiments were performed between *K. pneumoniae* KpSJTU083C1 to KpSJTU083C4 (donor) and *E. coli* HB101 (recipient). **B.** Growth curves of *E. coli* HB101, *E. coli* HB101::ICE*Kp1*, *E. coli* C600, and *E. coli* C600::ICE*Kp1*. **C.** Analysis of the stability of ICE*Kp1* within transconjugants *E. coli* HB101::ICE*Kp1* and *E. coli* C600::ICE*Kp1*.

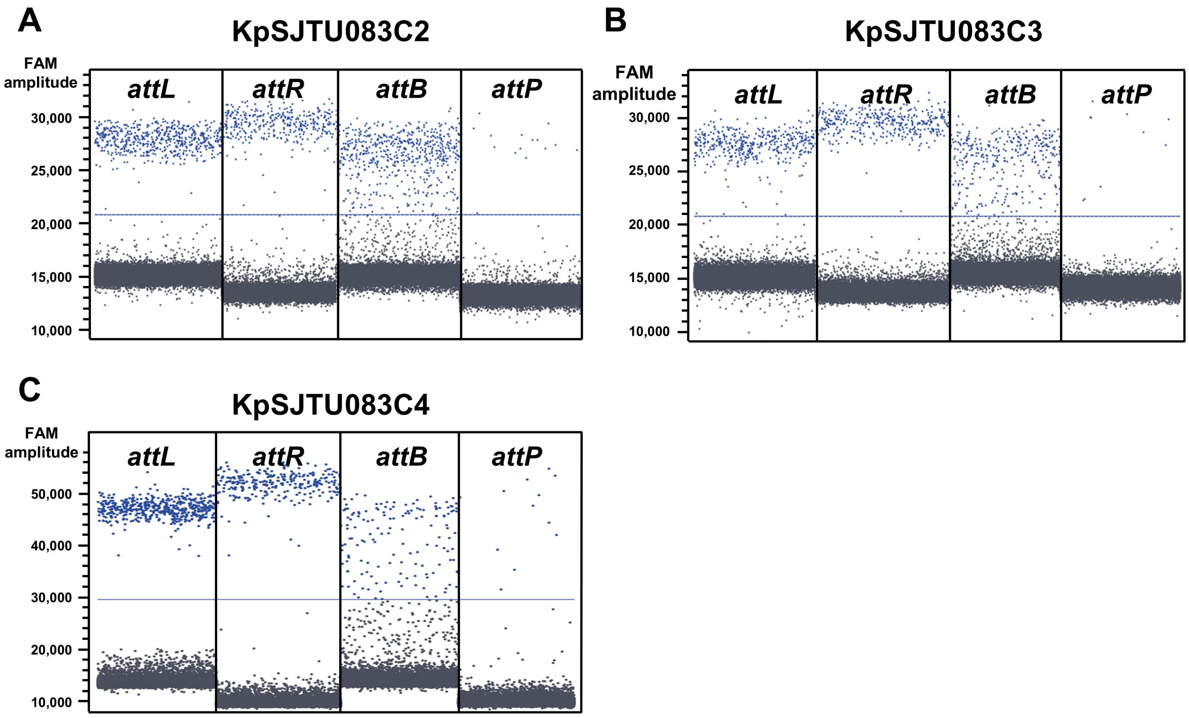

Figure S3. The 1D droplet spots of FAM fluorescence amplitude of *attL*, *attR*, *attB* and *attP* sites detected in KpSJTU083C2 (**A**), KpSJTU083C3 (**B**) and KpSJTU083C4 (**C**). Each point denotes a droplet, and those above the blue threshold line are classified as positive (blue points).

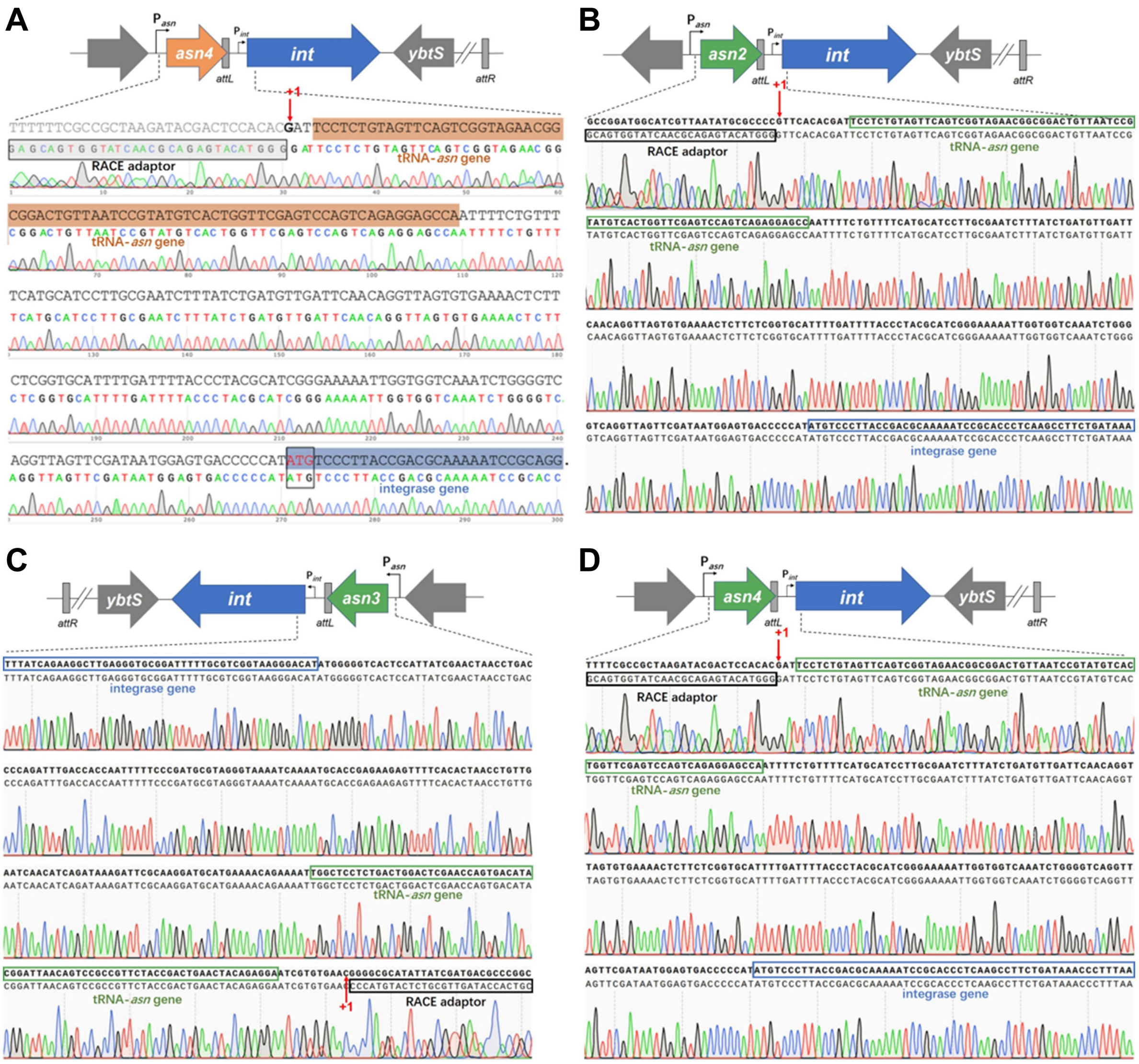

Figure S4. Transcription start site of the integrase gene identified in **(A)** NTUH-K2044, **(B)** KpSJTU083C2, **(C)** KpSJTU083C3 and **(D)** KpSJTU083C4 by 5'-RACE experiment. The sequence of the DNA fragment from the tRNA-*asn* promoter to the integrase gene (top) was aligned to the 5'-RACE-identified sequence (bottom). "+1" refers to the transcription start site of the integrase gene.

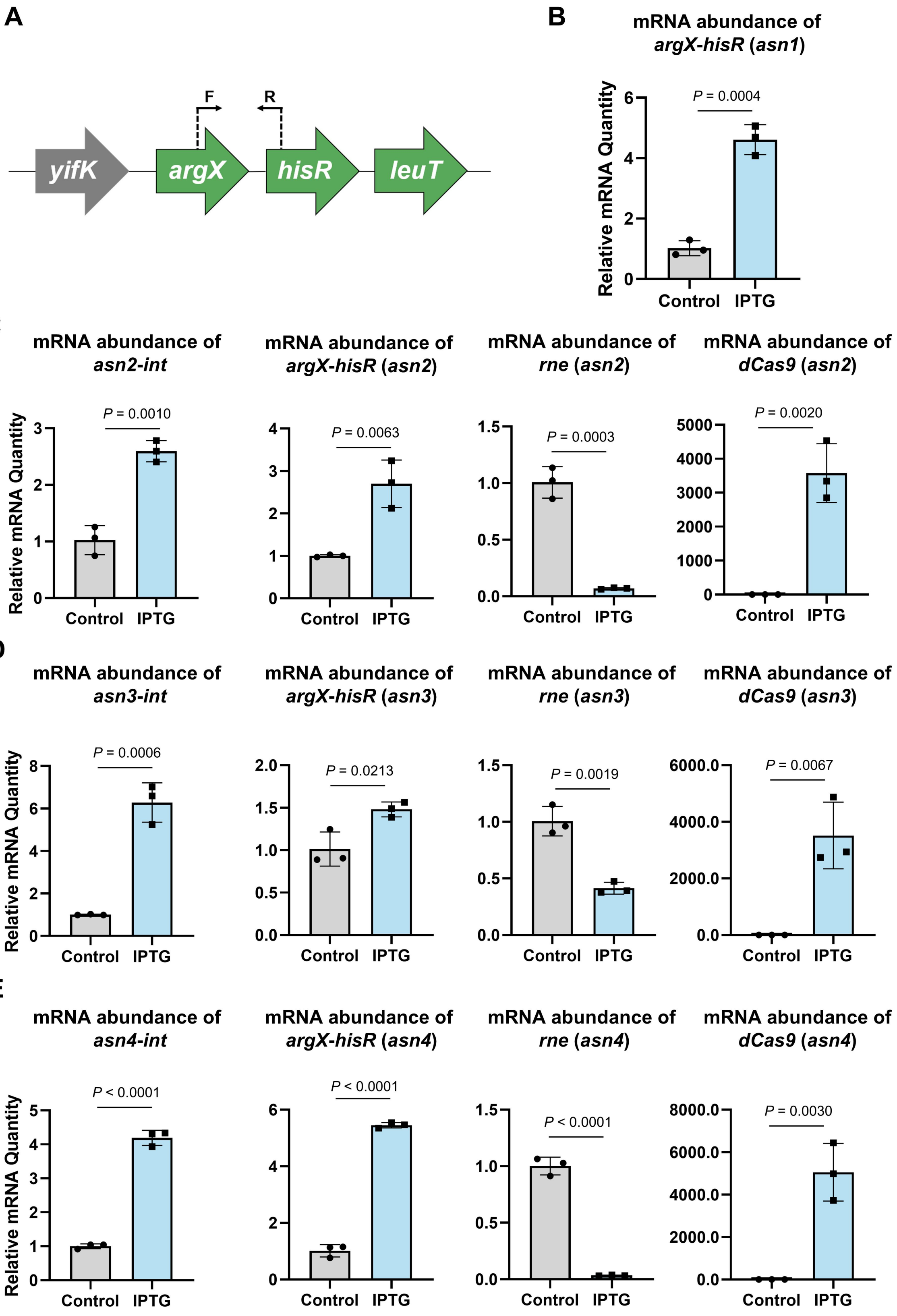

Figure S5. mRNA abundance of the junction sequence in the polycistronic transcripts KpSJTU083C1-KpSJTU083C4. **A.** Schematic map of the primer design for identification of the *argX-hisR* co-transcript cleavage. **B.** mRNA abundance of the *argX-hisR* transcript junction sequence in KpSJTU083C1. mRNA abundance of the junction sequence in the polycistronic transcripts of *asn-int* and *argX-hisR*, *rne* and *dCas9* in **(C)** KpSJTU083C2, **(D)** KpSJTU083C3 and **(E)** KpSJTU083C4. The mRNA quantity was normalized against the housekeeping gene *gapA*.

FigS6

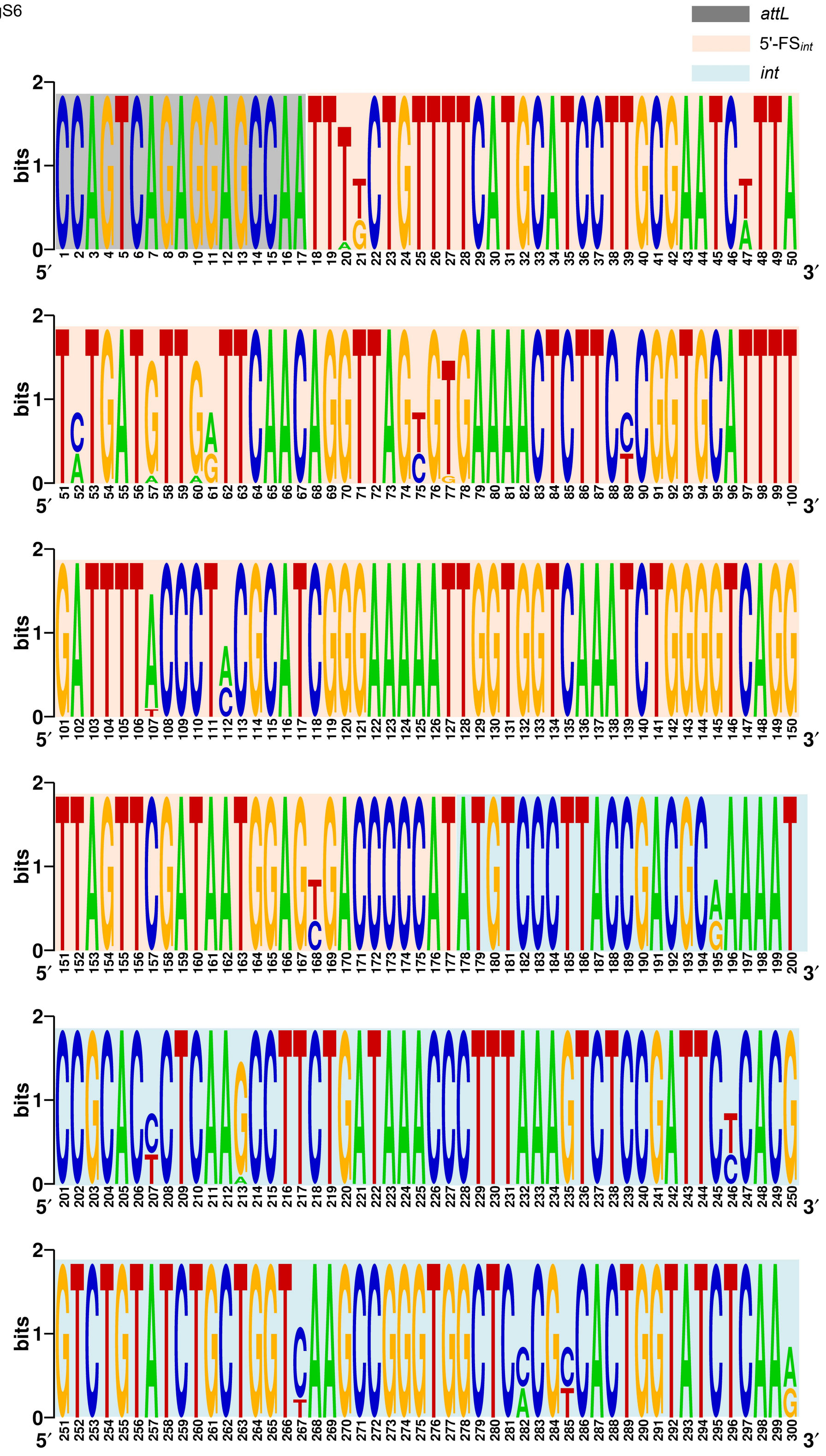

Figure S6. Sequence alignments and conservation analysis of the *attL* sites, the 5'-FS$_{int}$ and the partial sequences located at the 5' ends of integrase genes of fourteen ICE*Kp* families (ICE*Kp1*-ICE*Kp14*). The figure was constructed by WebLOGO.

FigS7

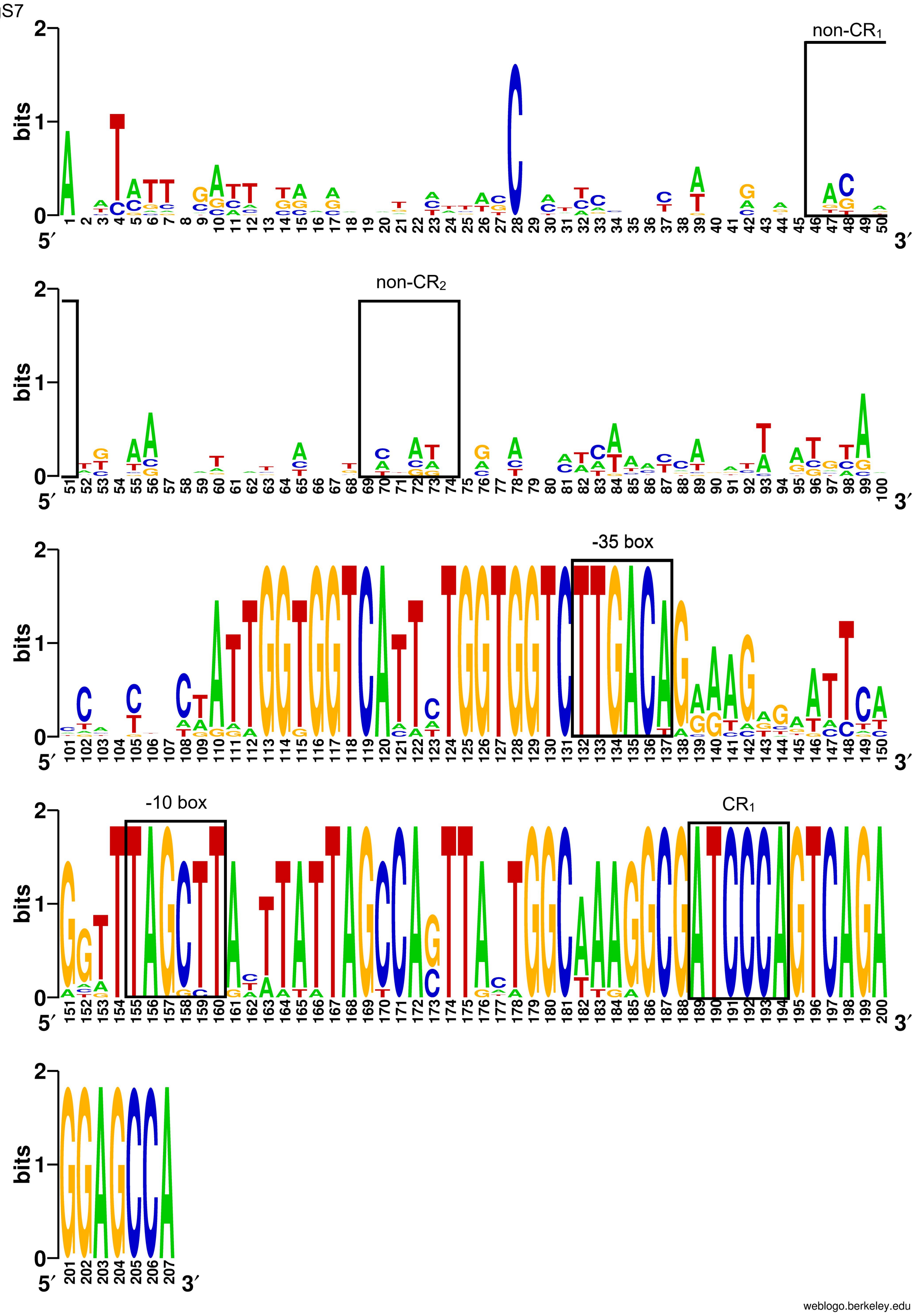

Figure S7. Sequence alignment and conservation analysis of the 3’ end of ICE*Kp* families (ICE*Kp1*-ICE*Kp14*). CR denotes conserved region. The figure was constructed by WebLOGO.

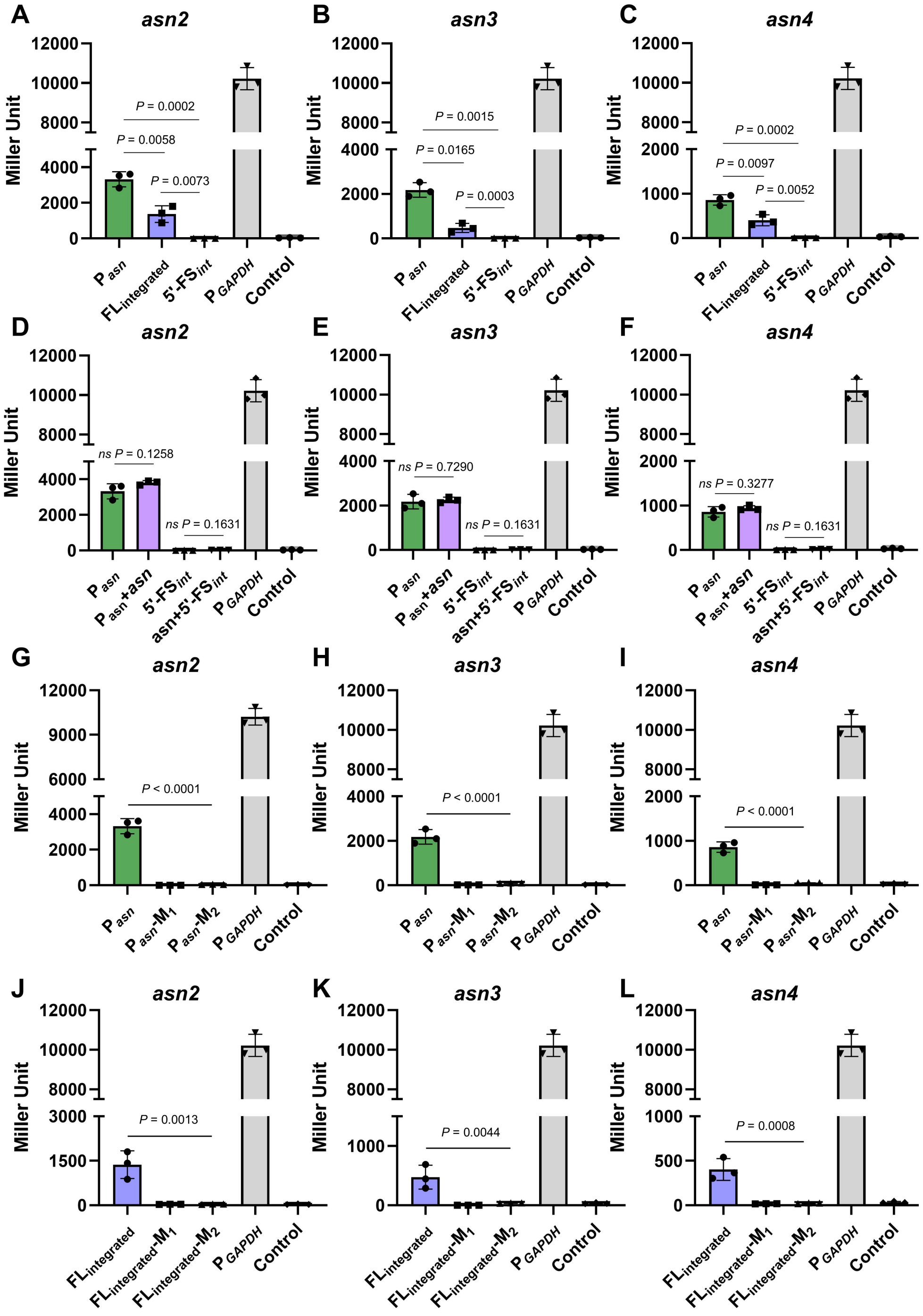

Figure S8. The promoter activity of the different upstream fragments of the integrase gene determined in the *asn2*, *asn3* and *asn4* groups by using the β-galactosidase assay. From the promoter of the tRNA-*asn* gene to the 5' end of the integrase gene, five groups were set: P_{asn} (tRNA-*asn* promoter), 5'-FS_{int} (the 5' flanking sequence of *int*), $FL_{integrated}$ (the full-length fragment covering tRNA-*asn* promoter, tRNA-*asn* and 5'-FS_{int}), P_{asn}+*asn* (tRNA-*asn* promoter and the tRNA-*asn* gene) and *asn*+5'-FS_{int} (the tRNA-*asn* gene and the 5'-FS_{int}). **A-C.** Promoter activities of the P_{asn}, FL, and 5'-FS_{int} groups when ICE*Kp1* integrated into the *asn2*, *asn3* and *asn4* sites by using the β-galactosidase assay. **D-F.** Promoter activities of the P_{asn}, P_{asn}+*asn*, 5'-FS_{int}, *asn*+5'-FS_{int} groups when ICE*Kp1* integrated into the *asn2*, *asn3* and *asn4* sites by using the β-galactosidase assay. The point-mutated promoter activity of the tRNA-*asn2* to tRNA-*asn4* **(G-I)** and their FL **(J-L)** groups determined by β-galactosidase assay. "-M_1" and "-M_2" refer to "point mutation at -10 box" and "point mutation at -35 box" of P_{asn2} –P_{asn4}, respectively.

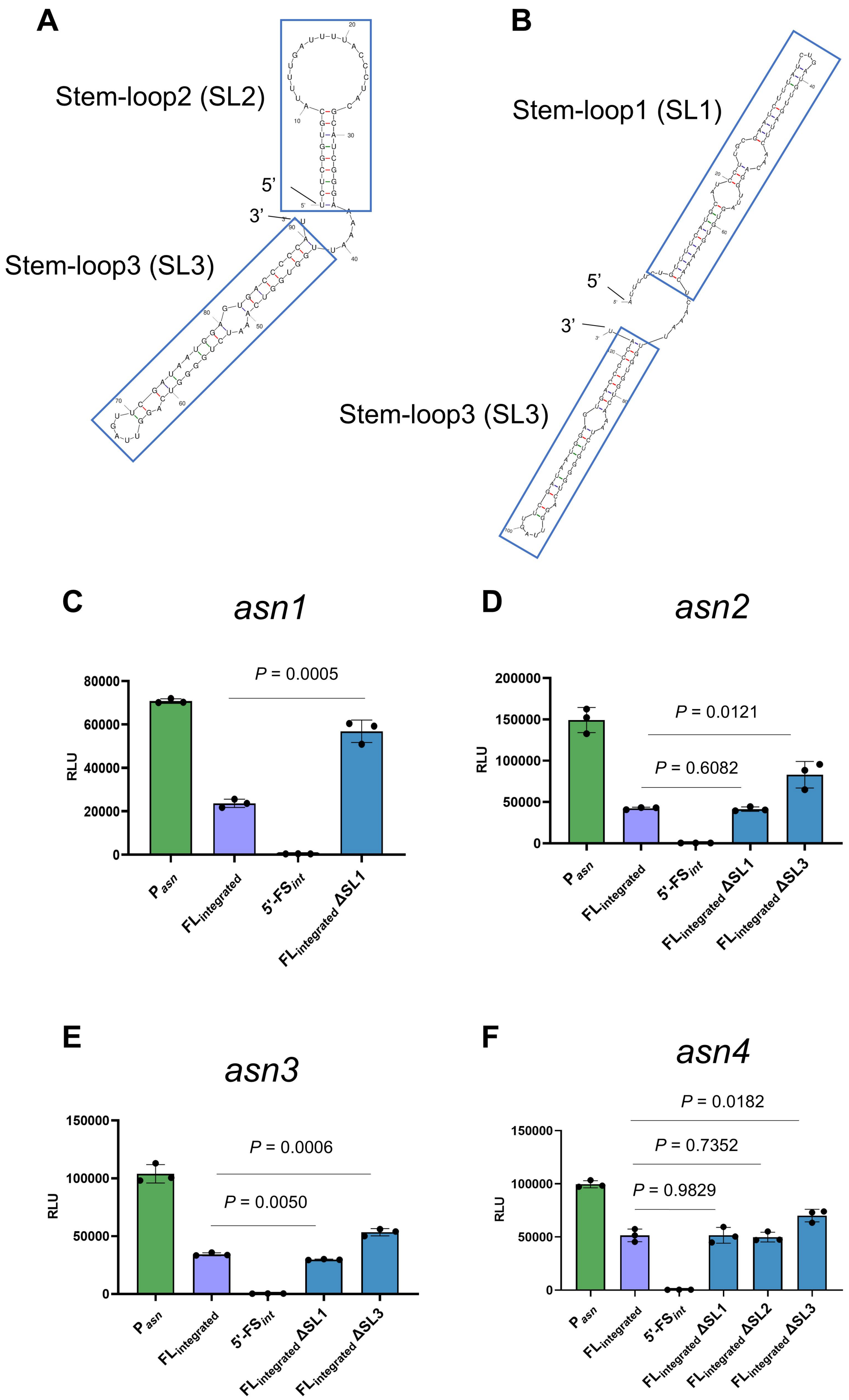

Figure S9. Detection of the effect of 5'-FS*int* stem-loop structures on promoter activity. Predicted mRNA secondary structure after knocking out the stem-loop1 **(A)** and stem-loop2 **(B)** in the 5' flanking region of ICE*Kp1*. The promoter activity changes of $FL_{integrated}$ after deletion of stem-loops in *asn1* **(C)**, *asn2* **(D)**, *asn3* **(E)** and *asn4* **(F)**.

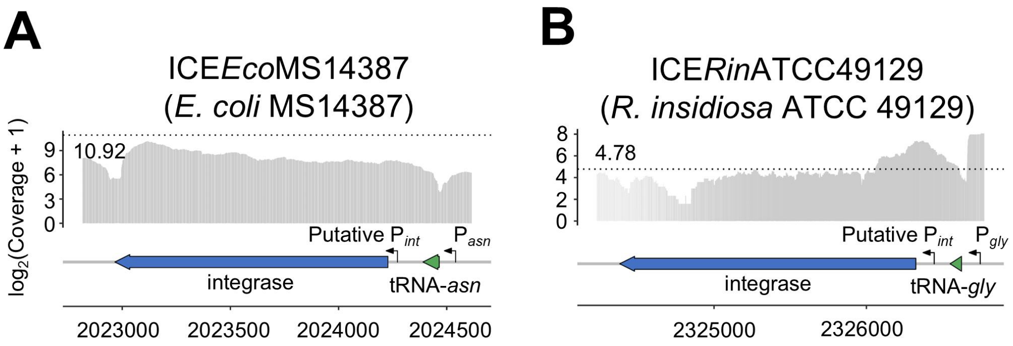

Figure S10. Analysis of the "*tRNA-int*" co-transcription in ICE*Ec1* and ICE*Ri1* by using RNA-seq data. The Y-axis represents the read coverage of the tRNA gene and the integrase gene in the RNA-seq data. The X-axis represents the gene locus of the tRNA gene and the integrase gene. The RNA-seq data were taken from NCBI SRA with the accession number of SRX8594985 for *E. coli* MS14387 and SRX12812169 for *Ralstonia insidiosa* ATCC 49129.

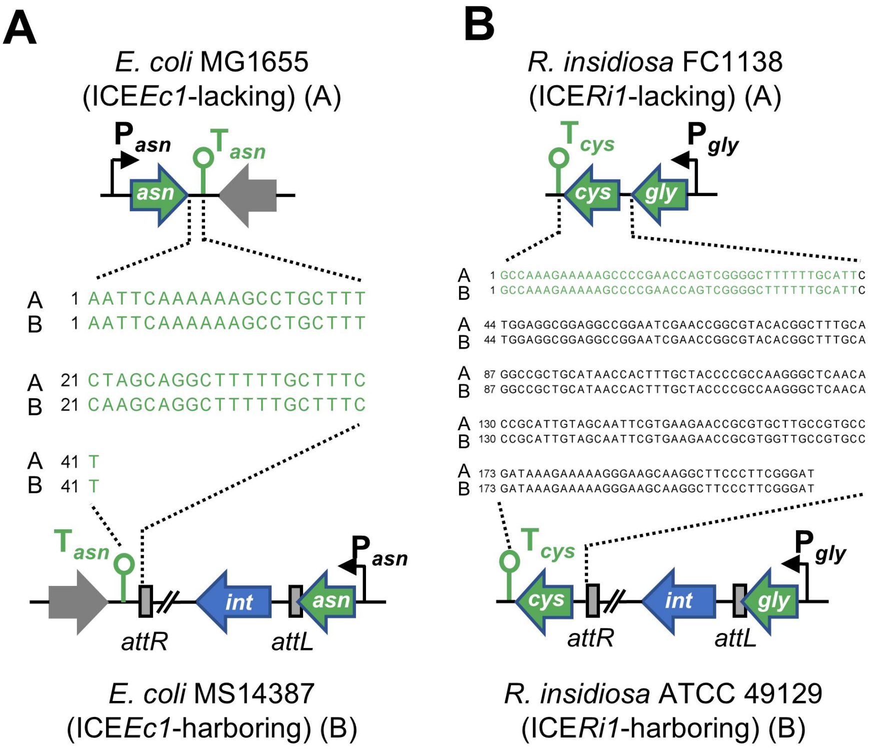

Figure S11. Location change event of the tRNA gene terminator after inserting different ICE families. Sequence alignment analysis of the sequence from the 3' end of the tRNA gene to the terminator before the insertion of ICE (top) and the sequence of the ICE 3' end after its insertion (bottom). The sequence in green denotes the terminator sequence of the tRNA gene.

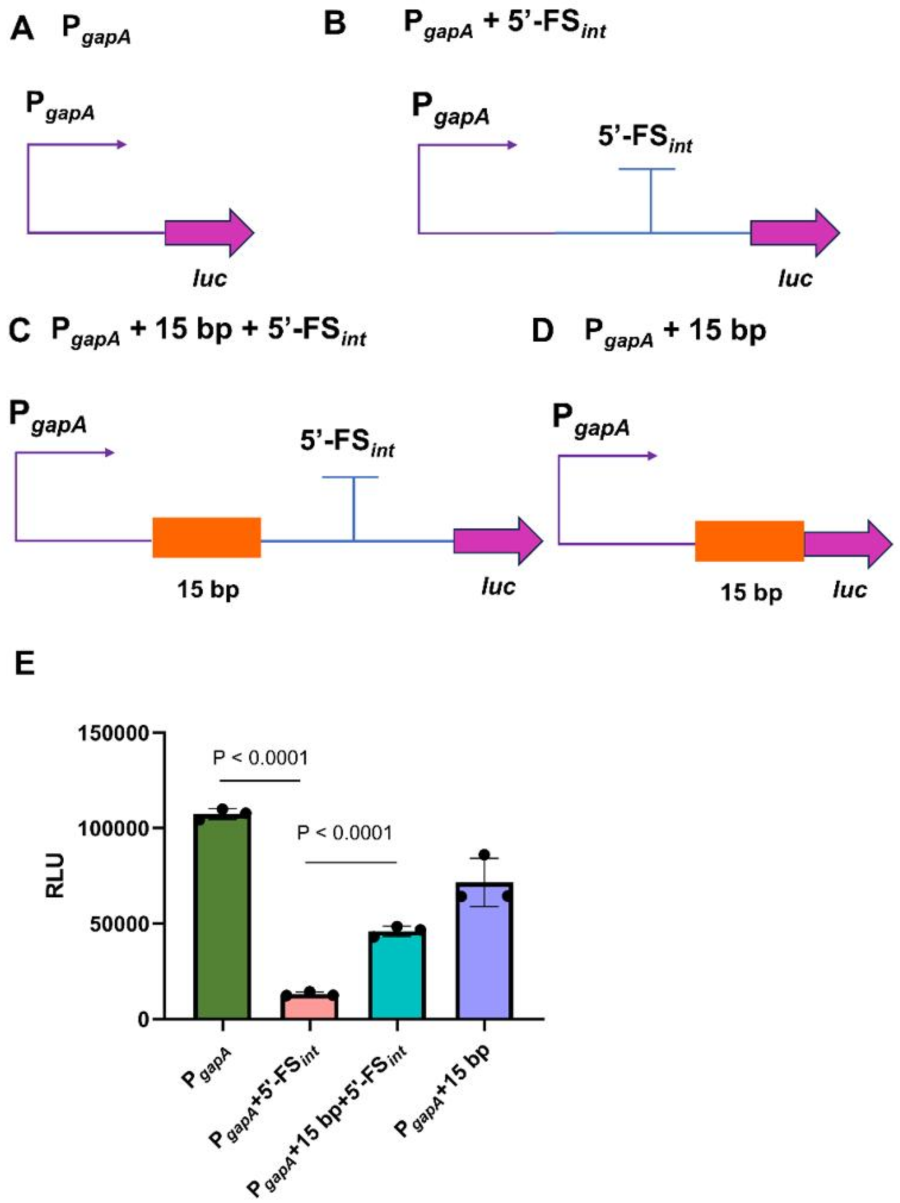

Figure S12. Effects of the FS_{int} and the 15 bp sequence of the ICE*Kp1* 3' end on the activity of the heterologous promoter P_{gapA}. **(A-D)** Schematic map of the luciferase reporter construct. P_{gapA} (the promoter of *gapA*), P_{gapA}+5'-FS_{int} (the *gapA* promoter and the 5' flanking sequence of *int*), P_{gapA}+15 bp+5'-FS_{int} (the *gapA* promoter, the 15 bp sequence from ICE*Kp1* 3'end and the 5' flanking sequence of *int*), P_{gapA}+15 bp (the *gapA* promoter and 15 bp sequence from ICE*Kp1* 3'end). **(E)** The promoter activity of P_{gapA}, P_{gapA}+5'-FS_{int}, P_{gapA}+15 bp+5'-FS_{int}, and P_{gapA}+15 bp.

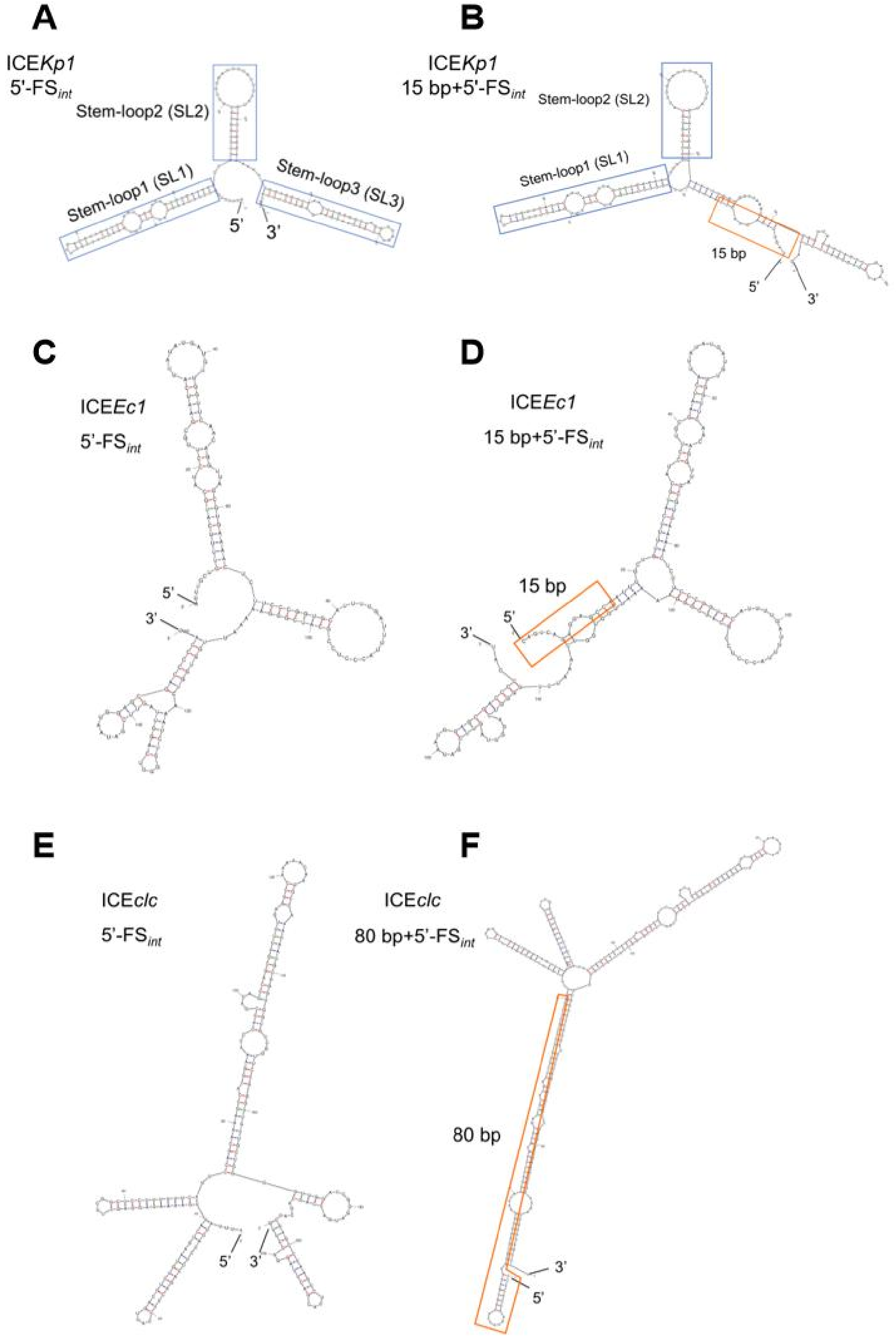

Figure S13. The predicted mRNA secondary structure of the 5'-FS*int* and 5' UTR of the *int* in excised ICE*Kp1* (**A** and **B**), ICE*Ec1* (**C** and **D**) and ICE*clc* (**E** and **F**). "15 bp" for ICE*Kp1* and ICE*Ec1* (or "80bp" for ICE*clc*) represents the DNA sequence from the transcription start site of *int* to the *attP* site.

Table S1. Strains and plasmids used in this study

Strain	Species	Genotype	Note	Source or Reference
NTUH-K2044	*K. pneumoniae*	ST23, K1 serotype, ICE*Kp1*$^{+}$	Clinical strain, isolated from a liver infection in Taiwan, China	(1)
NTUH-K2044IT	*K. pneumoniae*	ST23, K1 serotype, ICE*Kp1*$^{+}$	Derived from NTUH-K2044, donor in ICEKp1 conjugation experiment (NTUH-K2044IT/KpSJTU083-pAC YC184), HmR	This study
KpSJTU083	*K. pneumoniae*	ST15, KL24 serotype, ICE*Kp1*$^{-}$	Clinical strain, isolated from blood stream infections in Shanghai, China	This study
KpSJTU083A	*K. pneumoniae*	ST15, KL24 serotype, ICE*Kp1*$^{-}$	Derived from KpSJTU083, harbouring plasmid pACYC184, recipient in ICEKp1 conjugation experiment (NTUH-K2044IT/KpSJTU083-pAC YC184), AprR	This study
KpSJTU083C1	*K. pneumoniae*	Transconjugant NTUH-K2044/KpSJTU083-pAC YC184, ICEKp1+	Derived from KpSJTU083, harbouring ICEKp1 at asn1, model strain in this study	This study
KpSJTU083C2	*K. pneumoniae*	Transconjugant NTUH-K2044/KpSJTU083-pAC YC184, ICEKp1+	Derived from KpSJTU083, harbouring ICEKp1 at asn2, model strain in this study	This study
KpSJTU083C3	*K. pneumoniae*	Transconjugant NTUH-K2044/KpSJTU083-pAC YC184, ICEKp1+	Derived from KpSJTU083, harbouring ICEKp1 at asn3, model strain in this study	This study
KpSJTU083C4	*K. pneumoniae*	Transconjugant NTUH-K2044/KpSJTU083-pAC YC184, ICEKp1+	Derived from KpSJTU083, harbouring ICEKp1 at asn4, model strain in this study	This study
KpSJTU083C1::Tasn1	*K. pneumoniae*	Transconjugant NTUH-K2044/KpSJTU083-pAC YC184, ICEKp1+	Derived from KpSJTU083C1, knocked-in the terminator of asn1 at the 3' end of asn1 gene	This study
HB101	*E. coli*	*F*$^{-}$, *λ*$^{-}$,*araC14*, *leuB6(Am)*, *Δ(gpt-proA)62,lacY1*, *glnX44(AS)*, *galK2(Oc)*, *recA13*, *rpsL20(strR)*, *xyl A5*, *mtl-1*, *thiE1*, *[hsdS20]*	Recipient in ICEKp1 conjugation experiment (NTUH-K2044/HB101 and KpSJTU083C1-C4/HB101), SmR	(2)
HB101::ICE*Kp1*	*E. coli*	*F*$^{-}$, *λ*$^{-}$, *araC14*, *leuB6(Am)*, *Δ(gpt-proA)62*, *lacY1*, *glnX44(AS)*, *galK2(Oc)*, *recA13*, *rpsL20(strR)*, *xyl A5*, *mtl-1*, *thiE1*, *[hsdS20]*, ICE*Kp1*$^{+}$	Donor in ICE*Kp1* conjugation experiment (HB101::ICEKp1/C600),Hm^{R}, Sm^{R}	This study
C600	*E. coli*	*F*$^{-}$, *hsdR*, *lacY1*, *leuB6*, *supE44*, *thi-1*, *thr-1*, *tonA21*	Recipient in ICE*Kp1* conjugation experiment (HB101::ICE*Kp1*/C600), Rif^{R}	(3)
C600::ICE*Kp1*	*E. coli*	*F*$^{-}$, *hsdR*, *lacY1*, *leuB6*, *supE44*, *thi-1*, *thr-1*, *tonA21, ICEKp1+*	Transconjugant in ICEKp1 conjugation experiment (HB101::ICEKp1/C600), Hm^{R}, Rif^{R}	This study
DH5α	*E. coli*	*F*$^{-}$, *φ80*, *lacZΔM15*, *Δ(lacZYA-argF) U169*, *endA1*, *recA1*, *hsdR17(rk-,mk+)*, *supE44*, *λ*$^{-}$, *thi -1*, *gyrA96*, *relA1*, *phoA*	Tool strain used in plasmid conservation	Sangon Biotech
DH5α-pAClacZ	*E. coli*	*F*$^{-}$, *φ80*, *lacZΔM15*, *Δ(lacZYA-argF) U169*, *endA1*, *recA1*, *hsdR17(rk-,mk+)*, *supE44*, *λ*$^{-}$, *thi -1*, *gyrA96*, *relA1*, *phoA*	Derived from DH5α, habouring plasmid pAClacZ, used as the control group in β-galactosidase assay	This study
DH5α-pAClacZ-Pasn-K lebsiella pneumoniae KpSJTU083	*E. coli*	*F*$^{-}$, *φ80*, *lacZΔM15*, *Δ(lacZYA-argF) U169*, *endA1*, *recA1*, *hsdR17(rk-,mk+)*, *supE44*, *λ*$^{-}$, *thi -1*, *gyrA96*, *relA1*, *phoA*	Derived from DH5α, habouring plasmid pAClacZ-P_{asn}	This study
DH5α-pAClacZ-Pasn+a sn-Klebsiella pneumoniae KpSJTU083	*E. coli*	*F*$^{-}$, *φ80*, *lacZΔM15*, *Δ(lacZYA-argF) U169*, *endA1*, *recA1*, *hsdR17(rk-,mk+)*, *supE44*, *λ*$^{-}$, *thi -1*, *gyrA96*, *relA1*, *phoA*	Derived from DH5α, habouring plasmid pAClacZ-P_{asn}+*asn*	This study
DH5α-pAClacZ-FL-Kle bsiella pneumoniae KpSJTU083	*E. coli*	*F*$^{-}$, *φ80*, *lacZΔM15*, *Δ(lacZYA-argF) U169*, *endA1*, *recA1*, *hsdR17(rk-,mk+)*, *supE44*, *λ*$^{-}$, *thi -1*, *gyrA96*, *relA1*, *phoA*	Derived from DH5α, habouring plasmid pAClacZ-FL	This study
DH5α-pAClacZ-asn+P_{int}-Klebsiella	*E. coli*	*F*$^{-}$, *φ80*, *lacZΔM15*, *Δ(lacZYA-argF) U169*, *endA1*,	Derived from DH5α, habouring plasmid pAClacZ-asn+P_{int}	This study

pneumoniae KpSJTU083		*recA1*, *hsdR17(rk-,mk+)*, *supE44*, λ^-, *thi -1*, *gyrA96*, *relA1*, *phoA*		
DH5α-pAClacZ-P$_{int}$-Klebsiella pneumoniae KpSJTU083	*E. coli*	F^-, *φ80*, *lacZΔM15*, *Δ(lacZYA-argF) U169*, *endA1*, *recA1*, *hsdR17(rk-,mk+)*, *supE44*, λ^-, *thi -1*, *gyrA96*, *relA1*, *phoA*	Derived from DH5α, habouring plasmid pAClacZ-P$_{int}$	This study
DH5α-pAClacZ-PgapA-Klebsiella pneumoniae KpSJTU083	*E. coli*	F^-, *φ80*, *lacZΔM15*, *Δ(lacZYA-argF) U169*, *endA1*, *recA1*, *hsdR17(rk-,mk+)*, *supE44*, λ^-, *thi -1*, *gyrA96*, *relA1*, *phoA*	Derived from DH5α, habouring plasmid pAClacZ-P$_{gapA}$	This study
DH5α-pAClacZ-Pasn-Klebsiella pneumoniae NTUH-K2044	*E. coli*	F^-, *φ80*, *lacZΔM15*, *Δ(lacZYA-argF) U169*, *endA1*, *recA1*, *hsdR17(rk-,mk+)*, *supE44*, λ^-, *thi -1*, *gyrA96*, *relA1*, *phoA*	Derived from DH5α, habouring plasmid pAClacZ-P$_{asn}$	This study
DH5α-pAClacZ-FL-Klebsiella pneumoniae NTUH-K2044	*E. coli*	F^-, *φ80*, *lacZΔM15*, *Δ(lacZYA-argF) U169*, *endA1*, *recA1*, *hsdR17(rk-,mk+)*, *supE44*, λ^-, *thi -1*, *gyrA96*, *relA1*, *phoA*	Derived from DH5α, habouring plasmid pAClacZ-FL-NTUH-K2044	This study
DH5α-pAClacZ-P$_{asn}$-*Escherichia coli* ED1a	*E. coli*	F^-, *φ80*, *lacZΔM15*, *Δ(lacZYA-argF) U169*, *endA1*, *recA1*, *hsdR17(rk-,mk+)*, *supE44*, λ^-, *thi -1*, *gyrA96*, *relA1*, *phoA*	Derived from DH5α, habouring plasmid pAClacZ-P$_{asn}$-Escherichia coli ED1a	This study
DH5α-pAClacZ-FL-*Escherichia coli* ED1a	*E. coli*	F^-, *φ80*, *lacZΔM15*, *Δ(lacZYA-argF) U169*, *endA1*, *recA1*, *hsdR17(rk-,mk+)*, *supE44*, λ^-, *thi -1*, *gyrA96*, *relA1*, *phoA*	Derived from DH5α, habouring plasmid pAClacZ-FL-Escherichia coli ED1a	This study
DH5α-pAClacZ-P$_{int}$-*Escherichia coli* ED1a	*E. coli*	F^-, *φ80*, *lacZΔM15*, *Δ(lacZYA-argF) U169*, *endA1*, *recA1*, *hsdR17(rk-,mk+)*, *supE44*, λ^-, *thi -1*, *gyrA96*, *relA1*, *phoA*	Derived from DH5α, habouring plasmid pAClacZ-P$_{int}$-Escherichia coli ED1a	This study
DH5α-pAClacZ-3' end-*Escherichia coli* ED1a	*E. coli*	F^-, *φ80*, *lacZΔM15*, *Δ(lacZYA-argF) U169*, *endA1*, *recA1*, *hsdR17(rk-,mk+)*, *supE44*, λ^-, *thi -1*, *gyrA96*, *relA1*, *phoA*	Derived from DH5α, habouring plasmid pAClacZ-3' end-Escherichia coli ED1a	This study
DH5α-pAClacZ-Fusion P$_{3' end}$-*Escherichia coli* ED1a	*E. coli*	F^-, *φ80*, *lacZΔM15*, *Δ(lacZYA-argF) U169*, *endA1*, *recA1*, *hsdR17(rk-,mk+)*, *supE44*, λ^-, *thi -1*, *gyrA96*, *relA1*, *phoA*	Derived from DH5α, habouring plasmid pAClacZ-Fusion P$_{3' end}$-Escherichia coli ED1a	This study
DH5α-pAClacZ-P$_{gapA}$-*Escherichia coli* ED1a	*E. coli*	F^-, *φ80*, *lacZΔM15*, *Δ(lacZYA-argF) U169*, *endA1*, *recA1*, *hsdR17(rk-,mk+)*, *supE44*, λ^-, *thi -1*, *gyrA96*, *relA1*, *phoA*	Derived from DH5α, habouring plasmid pAClacZ-P$_{gapA}$-Escherichia coli ED1a	This study
DH5α-pAClacZ-P$_{gly}$-*Pseudomonas aeruginosa* W36662	*E. coli*	F^-, *φ80*, *lacZΔM15*, *Δ(lacZYA-argF) U169*, *endA1*, *recA1*, *hsdR17(rk-,mk+)*, *supE44*, λ^-, *thi -1*, *gyrA96*, *relA1*, *phoA*	Derived from DH5α, habouring plasmid pAClacZ-P$_{gly}$-Pseudomonas aeruginosa W36662	This study
DH5α-pAClacZ-FL-*Pseudomonas aeruginosa* W36662	*E. coli*	F^-, *φ80*, *lacZΔM15*, *Δ(lacZYA-argF) U169*, *endA1*, *recA1*, *hsdR17(rk-,mk+)*, *supE44*, λ^-, *thi -1*, *gyrA96*, *relA1*, *phoA*	Derived from DH5α, habouring plasmid pAClacZ-FL-Pseudomonas aeruginosa W36662	This study
DH5α-pAClacZ-P$_{int}$-*Pseudomonas aeruginosa* W36662	*E. coli*	F^-, *φ80*, *lacZΔM15*, *Δ(lacZYA-argF) U169*, *endA1*, *recA1*, *hsdR17(rk-,mk+)*, *supE44*, λ^-, *thi -1*, *gyrA96*, *relA1*, *phoA*	Derived from DH5α, habouring plasmid pAClacZ-P$_{int}$-Pseudomonas aeruginosa W36662	This study
DH5α-pAClacZ-3' end-*Pseudomonas aeruginosa* W36662	*E. coli*	F^-, *φ80*, *lacZΔM15*, *Δ(lacZYA-argF) U169*, *endA1*, *recA1*, *hsdR17(rk-,mk+)*, *supE44*, λ^-, *thi -1*, *gyrA96*, *relA1*, *phoA*	Derived from DH5α, habouring plasmid pAClacZ-3' end-Pseudomonas aeruginosa W36662	This study
DH5α-pAClacZ-Fusion P$_{3' end}$-*Pseudomonas aeruginosa* W36662	*E. coli*	F^-, *φ80*, *lacZΔM15*, *Δ(lacZYA-argF) U169*, *endA1*, *recA1*, *hsdR17(rk-,mk+)*, *supE44*, λ^-, *thi -1*, *gyrA96*,	Derived from DH5α, habouring plasmid pAClacZ-Fusion P$_{3' end}$-Pseudomonas aeruginosa W36662	This study

		relA1, *phoA*		
DH5α-pAClacZ-P_{gapA}-*Pseudomonas aeruginosa* W36662	*E. coli*	F^-, *φ80*, *lacZΔM15*, *Δ(lacZYA-argF) U169*, *endA1*, *recA1*, *hsdR17(rk-,mk+)*, *supE44*, λ^-, *thi -1*, *gyrA96*, *relA1*, *phoA*	Derived from DH5α, habouring plasmid pAClacZ-P_{gapA}-Pseudomonas aeruginosa W36662	This study
DH5α-pAClacZ-Fusion $P_{3'\,end}$-Pseudomonas aeruginosa W36662	*E. coli*	F^-, *φ80*, *lacZΔM15*, *Δ(lacZYA-argF) U169*, *endA1*, *recA1*, *hsdR17(rk-,mk+)*, *supE44*, λ^-, *thi -1*, *gyrA96*, *relA1*, *phoA*	Derived from DH5α, habouring plasmid pAClacZ-Fusion $P_{3'\,end}$-Pseudomonas aeruginosa W36662	This study
DH5α-pAClacZ-P_{int}	*E. coli*	F^-, *φ80*, *lacZΔM15*, *Δ(lacZYA-argF) U169*, *endA1*, *recA1*, *hsdR17(rk-,mk+)*, *supE44*, λ^-, *thi -1*, *gyrA96*, *relA1*, *phoA*	Derived from DH5α, habouring plasmid pAClacZ-P_{int}	This study
DH5α-pAClacZ-$P_{3'\text{-}end}$	*E. coli*	F^-, *φ80*, *lacZΔM15*, *Δ(lacZYA-argF) U169*, *endA1*, *recA1*, *hsdR17(rk-,mk+)*, *supE44*, λ^-, *thi -1*, *gyrA96*, *relA1*, *phoA*	Derived from DH5α, habouring plasmid pAClacZ-$P_{3'\text{-}end}$	This study
DH5α-pACYC184-Fusion $P_{3'\,end}$	*E. coli*	F^-, *φ80*, *lacZΔM15*, *Δ(lacZYA-argF) U169*, *endA1*, *recA1*, *hsdR17(rk-,mk+)*, *supE44*, λ^-, *thi -1*, *gyrA96*, *relA1*, *phoA*	Derived from DH5α, habouring plasmid pACYC184-Fusion $P_{3'\,end}$	This study
DH5α-pACYC184-P_{int}	*E. coli*	F^-, *φ80*, *lacZΔM15*, *Δ(lacZYA-argF) U169*, *endA1*, *recA1*, *hsdR17(rk-,mk+)*, *supE44*, λ^-, *thi -1*, *gyrA96*, *relA1*, *phoA*	Derived from DH5α, habouring plasmid pACYC184-P_{int}	This study
DH5α-pACYC184-$P_{3'\text{-}end}$	*E. coli*	F^-, *φ80*, *lacZΔM15*, *Δ(lacZYA-argF) U169*, *endA1*, *recA1*, *hsdR17(rk-,mk+)*, *supE44*, λ^-, *thi -1*, *gyrA96*, *relA1*, *phoA*	Derived from DH5α, habouring plasmid pACYC184-$P_{3'\text{-}end}$	This study
DH5α-pBluescript-5'+3'-int	*E. coli*	F^-, *φ80*, *lacZΔM15*, *Δ(lacZYA-argF) U169*, *endA1*, *recA1*, *hsdR17(rk-,mk+)*, *supE44*, λ^-, *thi -1*, *gyrA96*, *relA1*, *phoA*	Derived from DH5α, habouring plasmid pBluescript-5'+3'-int	This study

Plasmid	Genotype	Note	Source or Reference
pKOBEG	Expressing lambda Red genes *gam*, *bet* and *exo*	Temperature-sensitive, Apr^R	(4)
pFlp2	Expressing recombinase gene *flp*	Sucrose-sensitive, Apr^R	(5)
pACYC184	p15A origin of replication	Apr^R	(6)
pAClacZ	p15A origin of replication	Derived from pACYC184, cloned the the *E. coli* lacZ gene, cml^R	This study
pBluescript SK(+)	pUC origin of replication	high-copy-number, $spec^R$	(7)
pAClacZ-P_{asn}	p15A origin of replication	Derived from pAClacZ, cloned the promoter of the tRNA-asn gene, cml^R	This study
pAClacZ-P_{asn}+*asn*	p15A origin of replication	Derived from pAClacZ, cloned the promoter of the tRNA-asn gene and the sequence of the tRNA-asn gene, cml^R	This study
pAClacZ-FL	p15A origin of replication	Derived from pAClacZ, cloned the fragment from the promoter of the tRNA-*asn* gene to 5' end of the integrase gene, cml^R	This study
pAClacZ-*asn*+P_{int}	p15A origin of replication	Derived from pAClacZ, cloned the sequence of the tRNA-asn gene and the 161 bp intergenic region between the integrase gene and the tRNA-*asn* gene, cml^R	This study
pAClacZ-P_{int}	p15A origin of replication	Derived from pAClacZ, cloned the 161 bp intergenic region, cml^R	This study
pAClacZ-P_{gapA} -Klebsiella pneumoniae KpSJTU083	p15A origin of replication	Derived from pAClacZ, cloned the promoter sequence of *GAPDH*, cml^R	This study
pAClacZ-P_{asn}-Klebsiella pneumoniae NTUH-K2044	p15A origin of replication	Derived from pAClacZ, cloned the fragment the promoter of the tRNA-asn gene of NTUH-K2044, cml^R	This study
pAClacZ-FL-Klebsiella pneumoniae NTUH-K2044	p15A origin of replication	Derived from pAClacZ, cloned the fragment from the promoter of the tRNA-*asn* gene to 5' end of the integrase gene of NTUH-K2044, cml^R	This study
pAClacZ-P_{asn}-*Escherichia coli* ED1a	p15A origin of replication	Derived from pAClacZ, cloned the the promoter of the tRNA-*asn* gene of ED1a, cml^R	This study
pAClacZ-FL-*Escherichia coli*	p15A origin of replication	Derived from pAClacZ, cloned the fragment from the	This study

ED1a		promoter of the tRNA-*asn* gene to 5’ end of the integrase gene of ED1a, cml^R	
pAClacZ-P_{int}-*Escherichia coli* ED1a	p15A origin of replication	Derived from pAClacZ, cloned the 161 bp intergenic region of ED1a, cml^R	This study
pAClacZ-3' end-*Escherichia coli* ED1a	p15A origin of replication	Derived from pAClacZ, cloned the ICE*Ec1* 3' end of ED1a, cml^R	This study
pAClacZ-Fusion $P_{3' end}$-*Escherichia coli* ED1a	p15A origin of replication	Derived from pAClacZ, cloned the ICE*Ec1* Fusion $P_{3' end}$ fragment of ED1a, cml^R	This study
pAClacZ-P_{gapA}-*Escherichia coli* ED1a	p15A origin of replication	Derived from pAClacZ, cloned the promoter sequence of *GAPDH* of ED1a, cml^R	This study
pAClacZ-P_{gly}-*Pseudomonas aeruginosa* W36662	p15A origin of replication	Derived from pAClacZ, cloned the fragment from the promoter of the tRNA-*gly* gene to 5’ end of the integrase gene of W36662, cml^R	This study
pAClacZ-FL-*Pseudomonas aeruginosa* W36662	p15A origin of replication	Derived from pAClacZ, cloned the fragment from the promoter of the tRNA-*gly* gene to 5’ end of the integrase gene of W36662, cml^R	This study
pAClacZ-P_{int}-*Pseudomonas aeruginosa* W36662	p15A origin of replication	Derived from pAClacZ, cloned the 225 bp intergenic region of W36662, cml^R	This study
pAClacZ-$P_{3' end}$-*Pseudomonas aeruginosa* W36662	p15A origin of replication	Derived from pAClacZ, cloned the ICE*Ec1* 3' end of W36662, cml^R	This study
pAClacZ-Fusion $P_{3' end}$-*Pseudomonas aeruginosa* W36662	p15A origin of replication	Derived from pAClacZ, cloned the ICE*Ec1* Fusion $P_{3' end}$ fragment of W36662, cml^R	This study
pAClacZ-P_{gapA}-*Pseudomonas aeruginosa* W36662	p15A origin of replication	Derived from pAClacZ, cloned the promoter sequence of *GAPDH* of W36662, cml^R	This study
pAClacZ-Fusion $P_{3' end}$	p15A origin of replication	Derived from pAClacZ, cloned the ICE*Kp1* 3'-end and 274 bp intergenic region, cml^R	This study
pAClacZ-P_{int}	p15A origin of replication	Derived from pAClacZ, cloned the 274 bp intergenic region, cml^R	This study
pAClacZ-$P_{3'-end}$	p15A origin of replication	Derived from pAClacZ, cloned the ICE*Kp1* 3'-end, cml^R	This study
pBluescript-5'+3'-int	pUC origin of replication	Derived from pBluescript SK(+), cloned the ICE*Kp1* 3'+5'-int, spec^R	This study

Abbreviations: HmR, hygromycin resistance; AprR, apramycin resistance; SmR, streptomycin resistance; CmlR, chloramphenicol resistance; RifR, rifampicin resistance

Table S2. Primes used in this study

Primer	Sequence (5'-3')	Description
asn1-F (F_{tRIP})	TGGCGCGTAGACGTGAGAAT	Identification of the ICEKp1 insertion sites by tRIP-PCR
asn1-R (R_{tRIP})	CGCACTGGACAGGCTGTTTT	
asn2-F (F_{tRIP})	TGGGTCAGACTGCCGATATC	
asn2-R (R_{tRIP})	GCGCCAGGCTGGAGAAATAT	
asn3-F (F_{tRIP})	CGCTTTAATCTCAGCCGGGC	
asn3-R (R_{tRIP})	CTGGCTGTGGAAATATCCGC	
asn4-F (F_{tRIP})	GCACACGATAACGCTGACGT	
asn4-R (R_{tRIP})	GACGTTGCCGACGATAAGAG	
2044-IF	CCCTCGCGGTCAGTGAAAAT	Identification of the donor strain (NTUH-K2044) and recipient strain (KpSJTU083) in ICE*Kp1* conjugation assay
2044-IR	CGAAGCGCTGTTTGACGAAG	
KpSJTU083-IF	ATTTAGCGGCCCCTGCGGAA	
KpSJTU083-IR	TCCCGATCCTCGTAGCCTTC	
yhaI-F	GGAATGGCTTATTAAACACTGG	Identification of the donor strain (KpSJTU083::ICEKp1) and recipient strain (HB101 and C600) in ICEKp1 conjugation assay
yhaI-R	TTACGGTTTCTTCGACTGGT	
phoA-F	TCTGGAAATGGCAAAAGCCG	
phoA-R	GGATTTGGCGTACAGGTGAC	
bICE-VF	GGCTCATGGCATAAGGCTAT	
ICE-VR	TTGAAAATGCCAGCGGCCTA	
GSP-1	GATTACGCCAAGCTTAGCCATGCGCACGGTATTGCGCTCCTG	Determing the transcription start site of ICE*Kp1* integrase gene
GSP-2	GATTACGCCAAGCTTGCACTGCATGCCATTGCCCGGAAGCCG	
F0	GATTCCTCTGTAGTTCAGTCGG	Confirming the co-transcription of asn-integrase operon in KpSJTU083C1-C4 by RT-PCR
F1	CGTATGTCACTGGTTCGAGTCC	
F2	TTCATGCATCCTTGCGAATCTTT	
F3	GATGTTGATTCAACAGGTTAGTG	
F4	GTGAAAACTCTTCTCGGTGCAT	
F5	TTTTGATTTTACCCTACGCATCG	
F6	GAAAAATTGGTGGTCAAATCTGGG	
F7	TTACCGACGCAAAAATCCGCAC	
REV	TAAGGCGGTGCATAAGATTCTC	
RT-gapA-F	GGTATCAACGGTTTTGGCCG	Used as the control group in confirming the co-transcription of asn-integrase operon in KpSJTU083C1::Tasn1 by RT-PCR
RT-gapA-R	GGAGATGTGGGCAATCAGATCC	
qPCR-tRNA-F	CTGTAGTTCAGTCGGTAGAACGG	Confirming the junction sequence of asn-integrase operon in KpSJTU083C1-C4 by RT-qPCR
qPCR-IR-R	ATCAAAATGCACCGAGAAGAGTT	
qPCR-argX-F	CTCAGGTTCGAATCCTGTCGGG	Confirming the junction sequence of hisR-argX operon in KpSJTU083C1-C4 by RT-qPCR
qPCR-hisR-R	TCCAGGGCTCTACCAACTGAGC	
qPCR-rne-F	CTGAAGCACTGGGAAGCGAT	Determing the mRNA abundance of rne in KpSJTU083C1-C4 by RT-qPCR
qPCR-rne-R	TCAGGATTTCGCCGATGTCC	
qPCR-dCas9-F	ATCTCATTGCTCAGCTCCCC	Determing the mRNA abundance of dCas9 in KpSJTU083C1-C4 by RT-qPCR
qPCR-dCas9-R	GCATATTGATCTCCAATTTGCGCC	
qPCR-GAPDH-F	GCGTTGGAAACGATGTCCTG	Determing the mRNA abundance of gapA in KpSJTU083C1-C4 by RT-qPCR
qPCR-GAPDH-F	ACCGGTATCTTCCTGACCGA	
tRNA1-PonlyF	GTTAAATTGCCTCGAGCCTGTGGCAGCGTTTTTGCCACTT	Amplifying promoter region of tRNA-asn to construct plasmid pAClacZ-Pasn
tRNA1-PonlyR	AATTCCTCCTGGATCCATCGTGTGAACGAGGCGCAT	
tRNA2-PonlyF	GTTAAATTGCCTCGAGCAATTCGGTTTTTATTTGCCCGGT	
tRNA2-PonlyR	AATTCCTCCTGGATCCATCGTGTGAACGGGGCGCAT	
tRNA3-PonlyF	GTTAAATTGCCTCGAGTATTTCCGCATGGTGAGCGG	
tRNA3-PonlyR	AATTCCTCCTGGATCCATCGTGTGAACGGGGCGCAT	
tRNA4-PonlyF	GTTAAATTGCCTCGAGACGTTCAAGAGAGACCTTAG	
tRNA4-PonlyR	AATTCCTCCTGGATCCATCGTGTGGAGTCGTATCTT	
Ponly+tRNA-1F	CACAGTTAAATTGCCTCGAGCCTGTGGCAGCGTTTTTGCC	Amplifying promoter region of tRNA-asn and asn sequence to construct plasmid pAClacZ-Pasn+asn
Ponly+tRNA-1R	GGTGAATTCCTCCTGGATCCTGGCTCCTCTGACTGGACTC	
Ponly+tRNA-2F	CACAGTTAAATTGCCTCGAGCAATTCGGTTTTTATTTGCCCGG	
Ponly+tRNA-2R	GGTGAATTCCTCCTGGATCCTGGCTCCTCTGACTGGACTC	
Ponly+tRNA-3F	CACAGTTAAATTGCCTCGAGTATTTCCGCATGGTGAGCGG	
Ponly+tRNA-3R	GGTGAATTCCTCCTGGATCCTGGCTCCTCTGACTGGACTC	
Ponly+tRNA-4F	CACAGTTAAATTGCCTCGAGACGTTCAAGAGAGACCTTAGTGAG	
Ponly+tRNA-4R	GGTGAATTCCTCCTGGATCCTGGCTCCTCTGACTGGACTC	
Full length-1-F	CACAGTTAAATTGCCTCGAGCCTGTGGCAGCGTTTTTGCC	Amplifying from promoter region of tRNA-asn to 5'-end of integrase gene to construct plasmid pAClacZ-Pasn-FL
Full length-1-R	GGTGAATTCCTCCTGGATCCATGGGGGTCACTCCATTATCG	
Full length-2-F	CACAGTTAAATTGCCTCGAGCAATTCGGTTTTTATTTGCCCG	
Full length-2-R	GGTGAATTCCTCCTGGATCCATGGGGGTCACTCCATTATC	
Full length-3-F	CACAGTTAAATTGCCTCGAGTATTTCCGCATGGTGAGCGG	
Full length-3-R	GGTGAATTCCTCCTGGATCCATGGGGGTCACTCCATTATCG	

Full length-4-F	CACAGTTAAATTGCCTCGAGACGTTCAAGAGAGACCTTAGTG	
Full length-4-R	GGTGAATTCCTCCTGGATCCATGGGGGTCACTCCATTATC	
tRNA+IR-F	GTTAAATTGCCTCGAGTCCTCTGTAGTTCAGTCGGT	Amplifying from 5'-end of tRNA-asn to 5'-end of integrase gene to construct plasmid pAClacZ-asn+I.R.
tRNA+IR-R	GGTGAATTCCTCCTGGATCCATGGGGGTCACTCCATTATC	
I.R-F	GTTAAATTGCCTCGAGATTTGCTGTTTTCATGCATCCTTG	Amplifying from 3'-end of tRNA-asn to 5'-end of integrase gene to construct plasmid pAClacZ-I.R.
I.R-R	AATTCCTCCTGGATCCATGGGGGTCGCTCCATTATC	
F1	CCGACAGCATCCTTAAAGAG	Confirming the co-transcription of integrase operon in DH5α-pACYC184-Pcir by RT-PCR
F2	CTGCTAACAGTAACTTTCAGAC	
F3	ATCATCCTGTCCCTCCGTTA	
F4	ATCATCCCAGGAAAAGCATC	
F5	GGACTCTGAGGTGTCTGTTA	
F6	GATTGGTGGTCATTCTGGTG	
F7	CAGAGGAGCCAATTTTCTGTT	
F8	GTTCGATAATGGAGTGACCC	
F9	TTAGCGGTAAAGAATCCCGC	
R	TGCATAAGATTCTCGGCATG	
F_{attL}<br>F_{attB}	TAGAACGGCGGACTGTTAATCCG	Detection of the ICE*Kp1* excision in droplet digital PCR (ddPCR)
R_{attL}<br>R_{attP}	GCACCGAGAAGAGTTTTCACACTAACC	
F_{attR}<br>F_{attP}	GGTTTAGCTTACTTATTAGCCAGTTATTGG	
R_{attR1}<br>R_{attB1}	GCACCTACACCAGGATAAGATTGC	
R_{attR2}<br>R_{attB2}	TAAAGCCGCAATCGCCGGAGG	
R_{attR3}<br>R_{attB3}	GGATAAGCGAAACAAACGACCCG	
R_{attR4}<br>R_{attB4}	TTGTCAACGAGGTGCTTGCCAG	
Taqman-attL/B	TGTCACTGGTTCGAGTCCAGTCAGAGGAGCC	
Taqman-attR/P	CAAAGGCGATCCCAGTCAGAGGAGCCA	

Table S3. 23 ICEs which the integrase gene was located at the 5' end of the ICE and no other genes located between the tRNA gene and the integrase gene

ICE	Bacterial host	Insertion site	Intergenic sequence between tRNA and *int*	Coordinates	Integrase locus	tRNA locus	Accession number	SRA ID (RNA-seq data)	Reference
HPI-ICE*Eh1*	*Enterobacter hormaechei* 05-545	tRNA-*asn*	-	42411..108644	42589..43851	-	FN297818	-	(8)
ICE*Axy*A8-1	*Achromobacter xylosoxidans* A8	tRNA-*gly*	224 bp	1127770..1210913	1208744..1210687	1210912..1210987	CP002287	-	(9)
ICE*Bxe*LB400-1	*Burkholderia xenovorans* LB400	tRNA-*gly*	226 bp	3559420..3682252	3680095..3682026	3682253..3682328	CP000270	-	(10)
ICE*clc*	*Pseudomonas knackmussii* B13	tRNA-*gly*	226 bp	3709397..3812175	3810000..3811931	3812158..3812233	HG322950.1	-	unpublished
ICE*Dd1*	*Dickeya dadantii* 3937	tRNA-*asn*	161 bp	3126510..3201101	3126671..3127954	3126434..3126509	NC_014500	ERX5569329	(11,12)
ICE*Dda*Ech586-1	*Dickeya dadantii* Ech586	tRNA-*asn*	161 bp	2972027..3023093	2972189..2973460	2971952..2972027	CP001836	-	unpublished
ICE*Dze*Ech1591-1	*Dickeya zeae* Ech1591	tRNA-*asn*	161 bp	1679168..1733032	1731524..1732795	1732957..1733032	CP001655	-	unpublished
ICE*EcoI*HE3034-1	*Escherichia coli* IHE3034	tRNA-*asn*	161 bp	2192099..2246559	2192276..2193547	2192039..2192114	CP001969.1	-	(13)
ICE*Ec1*	*Escherichia coli* ED1a	tRNA-*asn*	161 bp	2164141..2223389	2164318..2165580	2164081..2164156	NC_011745	SRX5332356	unpublished
ICE*Eco*UMN026-1	*Escherichia coli* UMN026	tRNA-*asn*	161 bp	2277431..2343162	2277592..2278854	2277355..2277430	CU928163	-	unpublished
ICE*Hae*ULPAs1-1	*Herminiimonas arsenicoxydans*	tRNA-*gly*	226 bp	1978021..2066138	2063969..2065912	2066139..2066214	CU207211	-	(14)
ICE*Ml*Sym(R7A)	*Mesorhizobium japonicum* R7A	tRNA-*phe*	212 bp	5899546..6401363	5900184..5901428	5899896..5899971	NZ_CP051772	SRX8146967	(15)
ICE*Pae*690	*Pseudomonas aeruginosa* FFUP_PS_690	tRNA-*gly*	226 bp	315..86517	-	-	KY852375	-	unpublished
ICE*Pae*LESB58-1	*Pseudomonas aeruginosa* LESB58	tRNA-*gly*	226 bp	2796710..2907811	2796936..2798879	2796634..2796709	NC_011770	SRX6976232	(16,17)
ICE*Tau*DSM9187-1	*Tolumonas auensis* DSM 9187	tRNA-*gly*	226 bp	2621845..2718871	2716714..2718645	2718872..2718947	CP001616	-	(18)
PAGI-2	*Pseudomonas aeruginosa* C	tRNA-*gly*	228 bp	27286..132240	27514..29445	27210..27285	AF440523	-	(19)
ICE*Ec1*	*Escherichia coli* MS14387	tRNA-*asn*	161 bp	1965017..2024405	2022966..2024228	2024390..2024465	NZ_LR130564	SRX8594985	(20)
ICE*Ri1*	*Ralstonia insidiosa* strain ATCC 49129	tRNA-*gly*	224 bp	2212334..2326564	2324381..2326324	2326549..2326624	CP016022.1	SRX21061856	unpublished
ICE*Pps*CECT5344	*Pseudomonas pseudoalcaligenes* CECT 5344	tRNA-*gly*	236 bp	2014362..2117823	2014616..2016556	2014304..2014379	HG916826.1	SRX4408960	(21)
ICE*clc*	*Pseudomonas aeruginosa* W36662	tRNA-*gly*	225 bp	3486532..3601748	3599582..3601507	3601733..3601808	CP008870.2	SRX12812169	(22)
ICE*Ppu*UWC1	*Pseudomonas putida* UWC1	tRNA-*gly*	184bp	-	-	-	-	SRX13254565	(23)
CTnDOT	*Bacteroides thetaiotaomicron* VPI-5482	tRNA-*lys*	274 bp	2857562..2917821	2857851..2859111	2857505..2857577	AE015928.1	SRX20995037	unpublished

Table S4. *In silico* analysis of the ICE-inserted tRNA gene terminator in the ICE-harboring strains and the ICE-free strains

ICE	Strain	Accession number	ICE integration	ICE coordinates	ICE length	Insertion site	tRNA locus	tRNA gene terminator coordinates	Distance between the tRNA gene and its terminator
ICE*Kp1*	*Klebsiella pneumoniae* NTUH-K2044	AP006725.1	Y	3,395,820..3,472,027	76,208 bp	tRNA-*asn*	3,395,760-3,395,835	3,472,025-3,472,068	76,189 bp
	Klebsiella pneumoniae KpBSI083	-	N	-	-	-	1,788,753-1,788,828	1,788,711-1,788,754	-2 bp
ICE*Ec1*	*Escherichia coli* ED1a	NC_011745	Y	2,164,141..2,222,338	58,198 bp	tRNA-*asn*	2,164,081..2,164,156	2,222,417..2,222,467	58,260 bp
	Escherichia coli MG1655	U00096	N	-	-	-	2,044,549..2,044,624	2,044,703..2,044,753	78 bp
ICE*clc*	*Pseudomonas aeruginosa* W36662	CP008870.2	Y	3,486,532..3,601,748	115,217 bp	tRNA-*gly*	3,601,733..3,601,808	3,486,460..3,486,496	115,236 bp
	Pseudomonas aeruginosa PAO1	AE004091	N	-	-	-	2,923,222..2,923,297	2,923,150..2,923,186	35 bp
ICE*Ec1*	*Escherichia coli* MS14387	NZ_LR130564	Y	1,965,017..2,024,405	59,389 bp	tRNA-*asn*	2,024,390..2,024,465	1,964,977..1,965,017	59,372 bp
	Escherichia coli MG1655	U00096	N	-	-	-	2,062,260..2,062,335	2,062,336..2,062,376	0 bp
ICE*Ri1*	*Ralstonia insidiosa* ATCC 49129	CP016022.1	Y	2,212,334..2,326,564	114,231 bp	tRNA-*gly*	2,326,549..2,326,624	2,212,126..2,212,167	114,381 bp
	Ralstonia insidiosa FC1138	CP012605	N	-	-	-	2,334,429..2,334,504	2,334,220..2,334,261	167 bp

Reference:

1. Chou, H.-C., Lee, C.-Z., Ma, L.-C., Fang, C.-T., Chang, S.-C. and Wang, J.-T. (2004) Isolation of a chromosomal region of Klebsiella pneumoniae associated with allantoin metabolism and liver infection. *Infect Immun*, **72**, 3783-3792.
2. Boyer, H.W. and Roulland-Dussoix, D. (1969) A complementation analysis of the restriction and modification of DNA in Escherichia coli. *J Mol Biol*, **41**, 459-472.
3. Appleyard, R.K. (1954) Segregation of New Lysogenic Types during Growth of a Doubly Lysogenic Strain Derived from Escherichia Coli K12. *Genetics*, **39**, 440-452.
4. Chaveroche, M.K., Ghigo, J.M. and d'Enfert, C. (2000) A rapid method for efficient gene replacement in the filamentous fungus Aspergillus nidulans. *Nucleic Acids Res*, **28**, E97.
5. Hoang, T.T., Karkhoff-Schweizer, R.R., Kutchma, A.J. and Schweizer, H.P. (1998) A broad-host-range Flp-FRT recombination system for site-specific excision of chromosomally-located DNA sequences: application for isolation of unmarked Pseudomonas aeruginosa mutants. *Gene*, **212**, 77-86.
6. Bartolomé, B., Jubete, Y., Martínez, E. and de la Cruz, F. (1991) Construction and properties of a family of pACYC184-derived cloning vectors compatible with pBR322 and its derivatives. *Gene*, **102**, 75-78.
7. Alting-Mees, M.A. and Short, J.M. (1989) pBluescript II: gene mapping vectors. *Nucleic Acids Res*, **17**, 9494.
8. Paauw, A., Leverstein-van Hall, M.A., Verhoef, J. and Fluit, A.C. (2010) Evolution in quantum leaps: multiple combinatorial transfers of HPI and other genetic modules in Enterobacteriaceae. *PLoS One*, **5**, e8662.
9. Strnad, H., Ridl, J., Paces, J., Kolar, M., Vlcek, C. and Paces, V. (2011) Complete genome sequence of the haloaromatic acid-degrading bacterium Achromobacter xylosoxidans A8. *J Bacteriol*, **193**, 791-792.
10. Chain, P.S.G., Denef, V.J., Konstantinidis, K.T., Vergez, L.M., Agulló, L., Reyes, V.L., Hauser, L., Córdova, M., Gómez, L., González, M. *et al.* (2006) Burkholderia xenovorans LB400 harbors a multi-replicon, 9.73-Mbp genome shaped for versatility. *Proc Natl Acad Sci U S A*, **103**, 15280-15287.
11. Glasner, J.D., Yang, C.-H., Reverchon, S., Hugouvieux-Cotte-Pattat, N., Condemine, G., Bohin, J.-P., Van Gijsegem, F., Yang, S., Franza, T., Expert, D. *et al.* (2011) Genome sequence of the plant-pathogenic bacterium Dickeya dadantii 3937. *J Bacteriol*, **193**, 2076-2077.
12. Forquet, R., Jiang, X., Nasser, W., Hommais, F., Reverchon, S. and Meyer, S. (2022) Mapping the Complex Transcriptional Landscape of the Phytopathogenic Bacterium Dickeya dadantii. *mBio*, **13**, e0052422.
13. Moriel, D.G., Bertoldi, I., Spagnuolo, A., Marchi, S., Rosini, R., Nesta, B., Pastorello, I., Corea, V.A.M., Torricelli, G., Cartocci, E. *et al.* (2010) Identification of protective and broadly conserved vaccine antigens from the genome of extraintestinal pathogenic Escherichia coli. *Proc Natl Acad Sci U S A*, **107**, 9072-9077.
14. Muller, D., Médigue, C., Koechler, S., Barbe, V., Barakat, M., Talla, E., Bonnefoy, V., Krin, E., Arsène-Ploetze, F., Carapito, C. *et al.* (2007) A tale of two oxidation states: bacterial colonization of arsenic-rich environments. *PLoS Genet*, **3**, e53.
15. Ramsay, J.P., Bastholm, T.R., Verdonk, C.J., Tambalo, D.D., Sullivan, J.T., Harold, L.K., Panganiban, B.A., Colombi, E., Perry, B.J., Jowsey, W. *et al.* (2022) An epigenetic switch

activates bacterial quorum sensing and horizontal transfer of an integrative and conjugative element. *Nucleic Acids Res*, **50**, 975-988.

16. Pletzer, D., Sun, E., Ritchie, C., Wilkinson, L., Liu, L.T., Trimble, M.J., Wolfmeier, H., Blimkie, T.M. and Hancock, R.E.W. (2020) Surfing motility is a complex adaptation dependent on the stringent stress response in Pseudomonas aeruginosa LESB58. *PLoS Pathog*, **16**, e1008444.
17. Winstanley, C., Langille, M.G.I., Fothergill, J.L., Kukavica-Ibrulj, I., Paradis-Bleau, C., Sanschagrin, F., Thomson, N.R., Winsor, G.L., Quail, M.A., Lennard, N. *et al.* (2009) Newly introduced genomic prophage islands are critical determinants of in vivo competitiveness in the Liverpool Epidemic Strain of Pseudomonas aeruginosa. *Genome Res*, **19**, 12-23.
18. Chertkov, O., Copeland, A., Lucas, S., Lapidus, A., Berry, K.W., Detter, J.C., Del Rio, T.G., Hammon, N., Dalin, E., Tice, H. *et al.* (2011) Complete genome sequence of Tolumonas auensis type strain (TA 4). *Stand Genomic Sci*, **5**, 112-120.
19. Larbig, K.D., Christmann, A., Johann, A., Klockgether, J., Hartsch, T., Merkl, R., Wiehlmann, L., Fritz, H.-J. and Tümmler, B. (2002) Gene islands integrated into tRNA(Gly) genes confer genome diversity on a Pseudomonas aeruginosa clone. *J Bacteriol*, **184**, 6665-6680.
20. Mu, A., Klare, W.P., Baines, S.L., Ignatius Pang, C.N., Guérillot, R., Harbison-Price, N., Keller, N., Wilksch, J., Nhu, N.T.K., Phan, M.-D. *et al.* (2023) Integrative omics identifies conserved and pathogen-specific responses of sepsis-causing bacteria. *Nat Commun*, **14**, 1530.
21. Olaya-Abril, A., Luque-Almagro, V.M., Pérez, M.D., López, C.M., Amil, F., Cabello, P., Sáez, L.P., Moreno-Vivián, C. and Roldán, M.D. (2019) Putative small RNAs controlling detoxification of industrial cyanide-containing wastewaters by Pseudomonas pseudoalcaligenes CECT5344. *PLoS One*, **14**, e0212032.
22. Yan, J., Deforet, M., Boyle, K.E., Rahman, R., Liang, R., Okegbe, C., Dietrich, L.E.P., Qiu, W. and Xavier, J.B. (2017) Bow-tie signaling in c-di-GMP: Machine learning in a simple biochemical network. *PLoS Comput Biol*, **13**, e1005677.
23. Sulser, S., Vucicevic, A., Bellini, V., Moritz, R., Delavat, F., Sentchilo, V., Carraro, N. and van der Meer, J.R. (2022) A bistable prokaryotic differentiation system underlying development of conjugative transfer competence. *PLoS Genet*, **18**, e1010286.
